# Molecular Understanding of the Catalytic Consequence of Ketene Intermediates under Confinement

**DOI:** 10.1021/jacs.1c08036

**Published:** 2021-09-03

**Authors:** Wei Chen, Guangchao Li, Xianfeng Yi, Sarah J. Day, Karolina A. Tarach, Zhiqiang Liu, Shang-Bin Liu, Shik Chi Edman Tsang, Kinga Góra-Marek, Anmin Zheng

**Affiliations:** † State Key Laboratory of Magnetic Resonance and Atomic and Molecular Physics, National Center for Magnetic Resonance in Wuhan, Wuhan Institute of Physics and Mathematics, Innovation Academy for Precision Measurement Science and Technology, Chinese Academy of Sciences, Wuhan 430071, P. R. China; ‡ Faculty of Chemistry, Jagiellonian University in Krakow, Gronostajowa 2, Krakow 30-387, Poland; § Wolfson Catalysis Centre, Department of Chemistry, University of Oxford, Oxford OX1 3QR, United Kingdom; ∥ Diamond Light Source Ltd., Harwell Science and Innovation Campus, Didcot, OX11 0DE, United Kingdom; ⊥ Institute of Atomic and Molecular Sciences, Academia Sinica, Taipei 10617, Taiwan; # University of Chinese Academy of Sciences, Beijing 100049, P. R. China

## Abstract

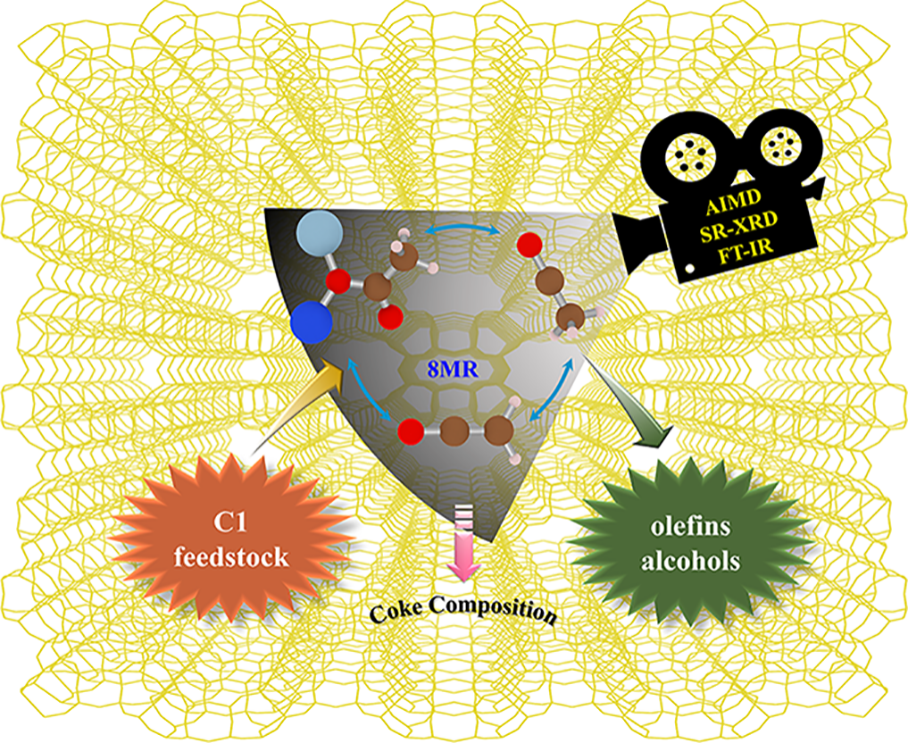

Neutral ketene is a crucial intermediate during zeolite carbonylation reactions. In this work, the roles of ketene and its derivates (viz., acylium ion and surface acetyl) associated with direct C–C bond coupling during the carbonylation reaction have been theoretically investigated under realistic reaction conditions and further validated by synchrotron radiation X-ray diffraction (SR-XRD) and Fourier transformed infrared (FT-IR) studies. It has been demonstrated that the zeolite confinement effect has significant influence on the formation, stability, and further transformation of ketene. Thus, the evolution and the role of reactive and inhibitive intermediates depend strongly on the framework structure and pore architecture of the zeolite catalysts. Inside side pockets of mordenite (MOR), rapid protonation of ketene occurs to form a metastable acylium ion exclusively, which is favorable toward methyl acetate (MA) and acetic acid (AcOH) formation. By contrast, in 12MR channels of MOR, a relatively longer lifetime was observed for ketene, which tends to accelerate deactivation of zeolite due to coke formation by the dimerization of ketene and further dissociation to diene and alkyne. Thus, we resolve, for the first time, a long-standing debate regarding the genuine role of ketene in zeolite catalysis. It is a paradigm to demonstrate the confinement effect on the formation, fate, and catalytic consequence of the active intermediates in zeolite catalysis.

## 1 Introduction

**Scheme 1:**
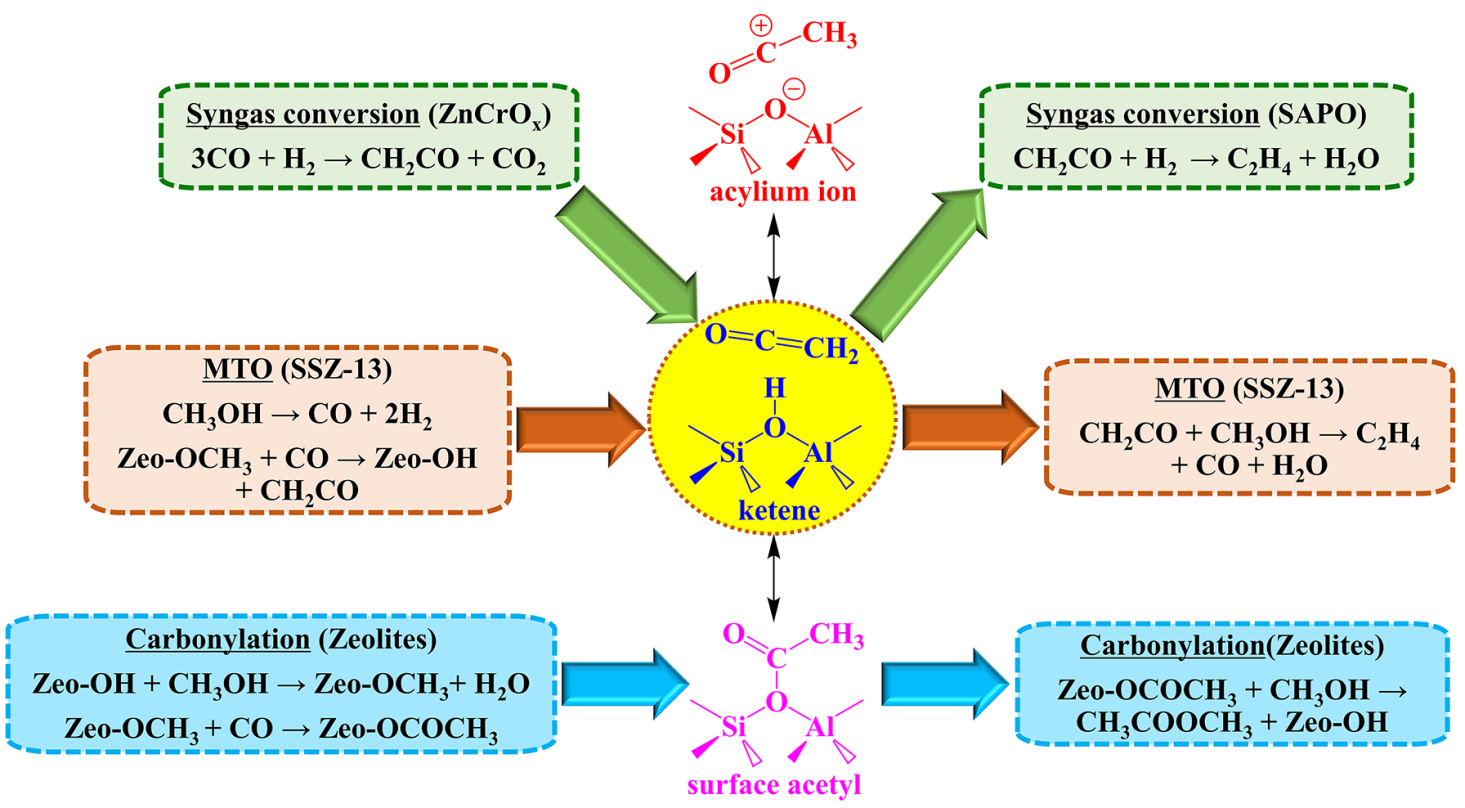
Mechanism of Direct C–C Bond Formation Associated with Interconversions of Ketene, Surface Acetyl, and Acylium Ion during Syngas Conversion, Carbonylation, and MTO Reactions over Zeolites

Ketenes, as one type of common active complexes in nucleophilic additions of organic synthesis,^1,2^ can also act as a key intermediate with the simplest formula (CH_2_=C=O) during zeolite-catalyzed C1 chemistry,^3^ such as methanol to olefins (MTO),^4,5^ dimethyl ether (DME) carbonylation to methyl acatate (MA),^6,7^ carbon dioxide to hydrocarbons,^8^ syngas conversion,^9,10^ and etc.^11,12^ (Scheme 1) For example, during the multicatalyst relay catalysis of oxide-zeolite for selective conversion of syngas to light olefins, ketene is produced from syngas over a metal oxide catalyst (e.g., ZnCrO_*x*_), which then diffuses into silicoaluminophosphate (SAPO) or MOR and further reacts with Brønsted acidic sites (BAS) to ultimately attain the desirable light olefins (C_2_^=^–C_4_^=^) selectivity under the shape selectivity of zeolites.^9,10^ Similar chemistry has been exploited for direct hydrogenation of CO_2_ to hydrocarbons^8^ by the bifunctional catalyst system comprising potassium superoxide doped iron oxide and acidic zeolites (e.g., ZSM-5 or MOR). Ketene generated from the deprotonation of surface acetyl over zeolitic catalyst has been shown to promote methylation and decarbonylation reactions during the MTO process.^4,5^ However, ketene is also known to give rise to catalyst deactivation via coke deposition.^13,14^ Evaluating the genuine role of ketene and its derivatives during catalytic reactions involving C1 feedstock is, therefore, a demanding but essential task.

More specifically, the stability and dynamic behavior of ketene and its derivatives in different channels could directly determine the activity and selectivity of C–C bond formation between surface methoxy species (SMS) and carbon monoxide (CO) during the carbonylation process. Zeolites (including metal-modified zeolites) and heteropolyacids are two types of solid acids particularly dedicated to catalyze DME/methanol carbonylation.^15,16^ Among these catalysts, acidic zeolites have been confirmed the high selectivity and conversion toward methyl acetate at relatively low temperatures because of their Brønsted acidity and pore architecture (Scheme S1). Distinctively, zeolites with 8MR channels, e.g., H-FER and H-MOR, were known to present an unprecedented molecular reactivity controlled by confinement to reaction process.^17−20^ Moreover, the unique acid site (T3-O33 position in 8MR channel of MOR) was considered to be the only active site for selective carbonylation as the transition state of C–C bond formation fit perfectly in the 8MR channel without the steric hindrance of methanol or DME.^21,22^ After C–C bond formation, surface acetyl, as one derivative of ketene, was commonly considered as the intermediate to produce MA and AcOH. Therefore, the possible stabilization or destabilization of above-mentioned unique confinement to ketene and its derivatives need to be analyzed because it is the important factor responsible for a remarkable catalytic activity offered by 8MR channel.

As the high active intermediate, ketene can easily convert into its two derivatives, namely, the protonated state of ketene (i.e., acylium ion, ionically interacted with the AlO_4_^–^ sites of delocalization, CH_3_CO^+^) and the rigid surface acetyl species (covalently bonded to the framework, Zeo–COCH_3_) commonly found over zeolitic catalysts. With an energy barrier of only ca. 17 kJ/mol, rapid equilibrium between ketene and surface acetyl may be reached over the MOR zeolite upon introducing D_2_O into deuterated acetic acid (CH_2_DCOOD) feedstock during the carbonylation reaction.^6^ Moreover, compared to these derivatives, the neutral form of ketene has a higher degree of freedom to transport or rotate in the pore channels of zeolites. However, its high reactivity and thermodynamic instability provokes further protonation and polymerization, which makes ketene difficult to capture by experimental techniques.^23−25^ In this context, the assessment of reactivity through a molecular dynamic study is the most useful way for understanding the roles of ketene during catalytic C1 reactions over zeolite-based catalysts.

Because of the instability and high activity, ketene and its derivatives are considered as the key-intermediates in different catalytic reactions but failed to be detected by experiments. Xie and co-workers^26^ investigated the role of ketene during direct conversion of syngas to light olefins over H-SAPO-34 zeolite. They concluded that the surface acetyl serves as the primary methylating agent toward hydrocarbon pool in zeotypes. Indeed, the relatively stable surface acetyl species are readily detected by solid-state ^13^C NMR and FT-IR spectroscopy as the intermediate during the MTO^27,28^ and DME/methanol carbonylation.^29,30^ Acylium ion (CH_3_CO^+^) may be formed during the acid-catalyzed reaction through interactions between the acylating agent (CH_3_COCl) and the Brønsted acid sites^31^ or via adsorption of CH_3_COCl in Lewis acidic AlCl_3_ catalyst.^32^ The surface acetyl, rather than acylium ion, have been reported as the reactive intermediate species in both Friedel–Crafts acylation over H-Beta and Koch-type carbonylation over H-MOR zeolites up to date.^33^ In this context, it is anticipated that acylium ion should be considered as highly active and short-living moiety over H-Beta and H-MOR zeolites, which makes its detection by *in situ* time-resolved spectroscopy very challenging.

In view of the significant role of ketene and its derivatives during catalytic C1 reactions over zeolite-based catalysts, this work aims to apply advanced *ab initio* molecular dynamic (AIMD) to trace the direct C–C bond coupling between SMS and CO in zeolites. By taking flexibility of zeolite framework and reaction temperature into consideration,^34−36^ it will be shown that the reaction mechanism associated with the formation of ketene and its derivatives during acid-catalyzed reaction over zeolitic catalysts can readily be obtained by AIMD simulations. Herein, H-MOR and H-SSZ-13, as two frequently mentioned zeolites related to ketene in MTO process, DME/MeOH carbonylation and syngas conversion, were considered as the model materials with 8MR channel or window topology to explore the influence of different zeolitic channels and cages to the evolution, stability, and electronic property of ketene and its derivatives. The SMS conversion in the 8MR channel of H-MOR (MOR-8MR) was followed to explore the specificity of the 8MR channel in the dynamical behavior of C–C bond formation and evolution of intermediates, whereas the SMS in 12MR channel of H-MOR (MOR-12MR) and CHA cage of H-SSZ-13 served as the reference internal voids. The intermediate chemistry of MTO process and syngas conversion in CHA topology of SSZ-13 was also proposed and examined. Finally, FT-IR and SR-XRD studies of CH_3_COCl transformation in MOR and H-SSZ-13 zeolites were used to validate the results of above AIMD simulations.

## 2 Results and Discussion

### 2.1 Dynamics of C–C Bond Coupling over Different Zeolites

**Figure 1:**
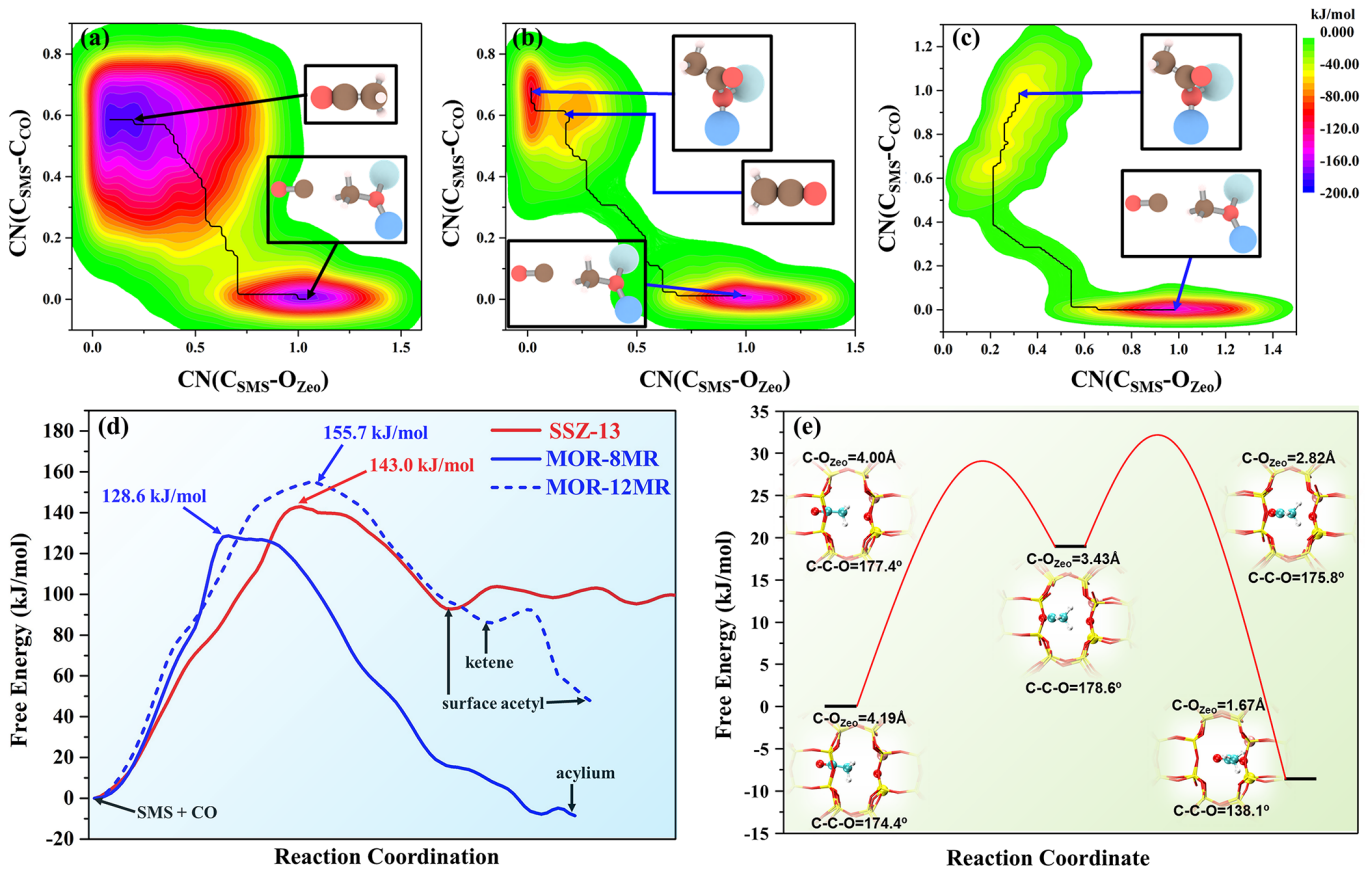
(a–c) Two-dimensional free energy profiles and primary product of C–C bond coupling between SMS and CO in (a) MOR-12MR, (b) MOR-8MR, and (c) SSZ-13 based on MTD-AIMD. The free energy profiles of SSZ-13 (Figure 1c) were reproduced based on our pervious results.^38^ CN(C_SMS_–O_Zeo_): coordination number between C(SMS) and O(AlO_4_), CN(C_SMS_–C_CO_): coordination number between C(SMS) and O(CO). The black lines represent the MFEP, which may be respectively projected as free energy surfaces displayed in d. (e) Free energy surfaces of acylium ion to surface acetyl in MOR-8MR. All the results are based on PBC model in Figure S1.

The rate-determining and pivotal step of DME/methanol carbonylation and MTO initiation via CO pathway over zeolites is the C–C bond coupling between SMS and CO. As displayed in Figures 1a–c, the 2D free energy profiles obtained from MOR-8MR, MOR-12MR, and SSZ-13 all consist of two basins arising from the reactants (bottom right) and products (upper left), respectively. Moreover, it was found that direct C–C bond couplings were associated with C_SMS_–O_Zeo_ bond ruptures and C_SMS_–C_CO_ bond formations reflected by their coordination numbers (CN). It is also noteworthy that primary products in the product basin are rather different within the different framework structures and pore channels of zeolites. Surface acetyl and ketene were found in MOR-12MR, whereas the acylium ion was found in MOR-8MR and surface acetyl was identified in SSZ-13 as the only species. The corresponding free energy surfaces projected from minimum free energy paths (MFEP) are displayed in Figure 1d. The free energy barriers observed for MOR-12MR, MOR-8MR, and SSZ-13 were found to be 155.7, 128.6, and 143.0 kJ/mol, and these results are basically consistent with the static DFT calculations and pervious theoretical studies, as summarized in Tables S1 and S2.^7,20−22,37^ Notably, it is incontrovertible that MOR-8MR has a significantly higher activity on C–C bond coupling than do MOR-12MR and SSZ-13, but the order of activity between MOR-12MR and SSZ-13 is indistinguishable by static DFT calculations because both MOR-12MR and SSZ-13 are loosely confined by the reaction process. This evidence shows that the activation of SMS and the C–C bond coupling, where the latter is directly related to the reaction rate, selectivity, and yield of MA and AcOH, is more pronounced for the carbonylation reaction in MOR-8MR when compared to that of MOR-12MR and SSZ-13. The MFEP (Figure 1d) also revealed that acylium ion was highly stabilized inside the confined side pocket of MOR-8MR, whereas the surface acetyl is stable inside MOR-12MR and SSZ-13. Such a discrepancy in the nature of the products stabilized inside diverse internal voids implies that the transformation of the acylium ion to surface acetyl involved two different free energy barriers with the maximum free energy span of 40.2 kJ/mol. (Figure 1e) One is the barrier to overcome the mobility of the acylium ion from the side pocket to the 8MR channels, whereas the other should be the reconfiguration barrier for the formation of surface acetyl. This free energy span originating from the localized confinement and steric hindrance maintains the kinetic stability of the acylium ion in MOR-8MR. Furthermore, it is intriguing that the presence of a metastable ketene intermediate was found only in the MOR-12MR and not in SSZ-13 as either a stable or metastable species. Therefore, it is highly desirable to explore the possible short-living intermediates during the evolution of direct C–C bond coupling in MOR and SSZ-13 to provide deeper insight into the unique role of the zeolitic framework 8-MR topology.

**Figure 2:**
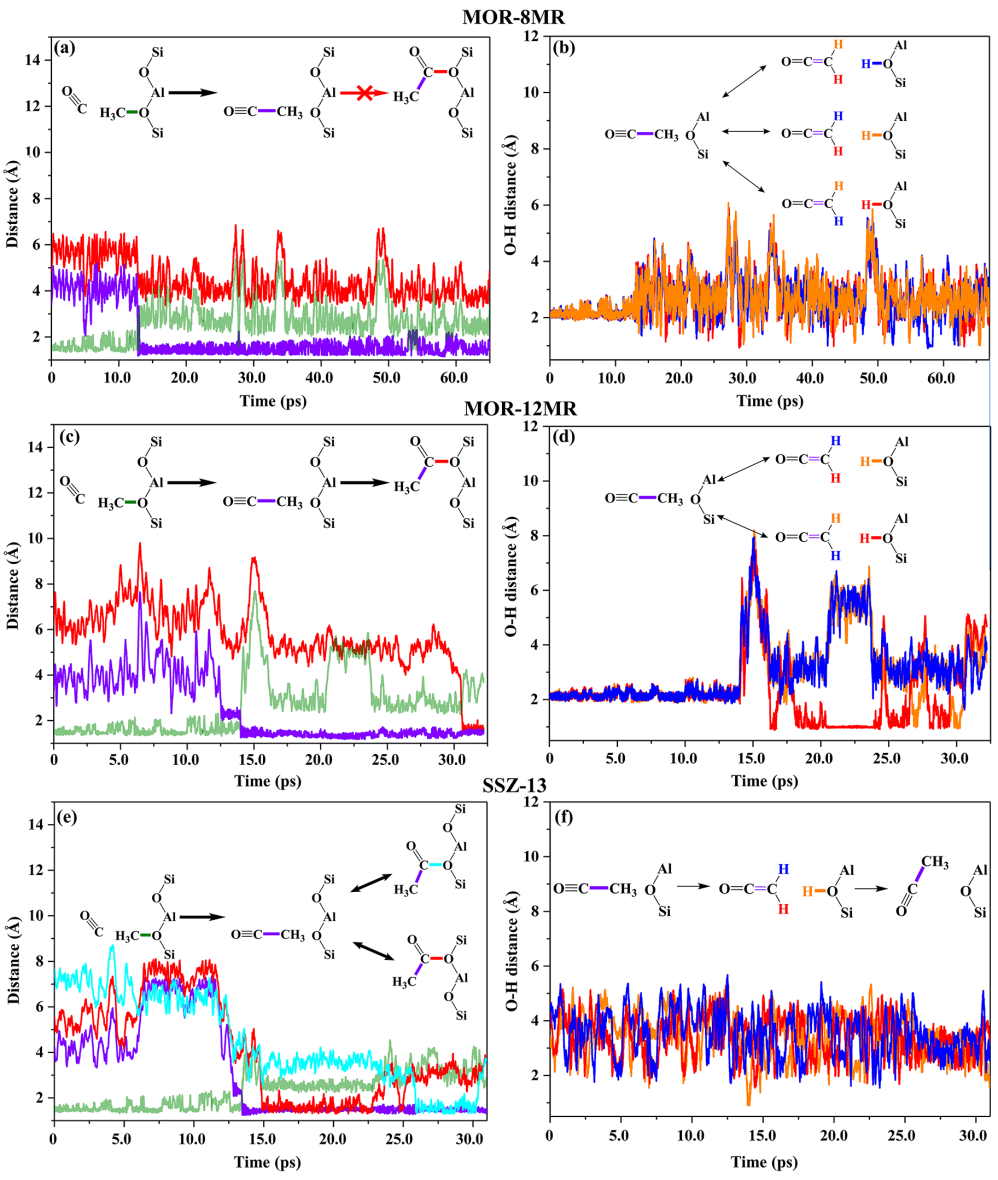
Critical distance evolutions of direct C–C bond coupling between Zeo–OCH_3_ and CO at 473 K in (a, b) MOR-8MR, (c, d) MOR-12MR, and (e, f) SSZ-13. The C–C/C–O bond distance evolution of SSZ-13 (Figure 2e) were reproduced by our pervious results.^38^ The evolutions of C_SMS_–O_Zeo_, C_SMS_–C_CO_, and C_CO_–O_Zeo_ distances are depicted in a, c, and e, whereas that of O_Zeo_–H_SMS_ distances are shown in b, d, and f. The colors of curves are consistent with the color in embedded chemical formulas. Herein, the time of AIMD simulations in horizontal axis was not corresponded to the real time of reaction, which were accelerated by the MTD method.

It is noteworthy that the dynamic behaviors in the time scale (picosecond) of MTD accelerated AIMD simulations (MTD-AIMD) cannot real-time reflect the reaction process in the time scale (second) of the reaction; thus the dynamic evolution of assorted bond distances would be helpful to understand the dynamic reaction process and capture some metastable intermediates in different zeolites as depicted in Figure 2 and visualized in Movies S1–S3. The formation of the C–C bond (purple curve) is seen at ca. 12.8, 14.0, and 13.3 ps in MOR-8MR (Figure 2a), MOR-12MR (Figure 2c), and SSZ-13 (Figure 2e), respectively, with C–C distance smaller than 1.5 Å. Notably, there are interrupted time slots before the C–C bond formation as the collision attempts, and the stronger constraint of MOR-8MR to reactants led to fewer ineffective collisions than these of MOR-12MR and SSZ-13. The MOR-8MR, which has more confined geometry than MOR-12MR and SSZ-13, clearly facilitates C–C bond formation. In addition, a notable decrease in the C_SMS_–C_CO_ bond distance (from ca. 2.8 Å to ca. 2.0 Å) was observed because of the activation of SMS and CO. It has been illustrated that such an activated state was provoked by repeated collisions of SMS and CO through the adjustment of molecular configuration and velocity of the collision during the reaction.^39,40^ Therefore, it is envisaged that a more sophisticated process is involved for C–C bond coupling in MOR-12MR and SSZ-13 than that in MOR-8MR. A process in SSZ-13 and MOR is proposed for the formation of surface acetyl through C–O bond coupling in Figure 2c–f (the SSZ-13 as an example, Figure S2), which is realized in the five-step route involving C–C bond coupling, deprotonation, rotation, protonation, and finally the C–O bond formation.

**Scheme 2:**
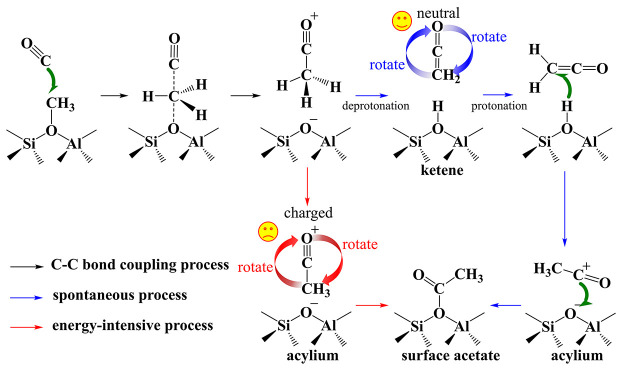
Routes Leading to Surface Acetyl in MOR and SSZ-13 with and without Participation of Ketene: Pathway in MOR-12MR and SSZ-13 (Blue Arrows) and in MOR-8MR (Red Arrows) According to Figure 1e

Besides monitoring the reaction process at reaction condition, the other advantage of AIMD is the capturing all stable as well as metastable species through the observing their structural evolution. In contrast to static DFT calculations, by which the formation of surface acetyl species is directly predicted without the participation of ketene,^20,22,41^ acylium ion, and ketene were also detected as intermediates prior to the formation of surface acetyl in the case of AIMD (Figure 2). On the basis of free energy surface analysis, the most energy-intensive step is the rotation of the acylium ion to bond directly with the oxygen atom on the framework AlO_4_^–^ tetrahedron through strong electrostatic interactions. Thus, as illustrated in Scheme 2, ketene serves as the key intermediate in MOR-12MR and SSZ-13 to significantly reduce the detrimental interactions. Hence, the formation of neutral ketene (via deprotonation of acylium ion) is inevitable to avoid the energy barrier arising from strong electrostatic interactions during rotation, while also being favorable for the formation of surface acetyl species. The above notion is in line with experimental results reported by Jensen and co-workers.^6,20^

As ketene intermediates were readily observed in MOR-12MR and SSZ-13 during the carbonylation reaction, this again demonstrates that AIMD simulations are indeed capable of differentiating subtle differences in the structural framework of zeolites, hence the duration and genuine role of ketenes during catalytic reactions were worth further exploring. In MOR-12MR, the metastable ketene intermediate, which was generated immediately after the formation of C–C bond, persisted for a rather long time (16.2–30.3 *ps*; see variations of the O_Zeo_–H_SMS_ bond curve in Figure 2d). During this period, ketene constantly adjusts its orientation until the eventual formation of surface acetyl. By comparison, ketene lasted for a relatively shorter time (13.8–14.2 *ps*; see Figure 2f) before it was protonated to form surface acetyl in SSZ-13. On the other hand, in the narrower pore channels of MOR-8MR, triggered by simultaneous deprotonation and reprotonation starting at ca. 29 ps, the cyclic appearance/disappearance of ketene was observed. Consequently, a dynamic equilibrium between ketene and acylium ion was reached, in which the equilibrium leaned toward acylium ion (>95%). Apparently, the presence of a ketene intermediate in MOR-12MR and SSZ-13 is mainly due to the more facile adjustment of its orientation, leading to the formation of surface acetyl. Nevertheless, it is noteworthy that a prolonged duration of ketene in MOR-12MR is detrimental as it promotes deactivation of the catalyst via oligomerization reactions^42^ and formation of carbonaceous deposits.^13,14^ It has been shown that ketene may also be formed during C–C bond coupling regardless of the formation pathway via deprotonation of surface acetyl during the MTO process.^4,5^

### 2.2 Electronic Evolutions of C–C Bond Coupling

**Figure 3:**
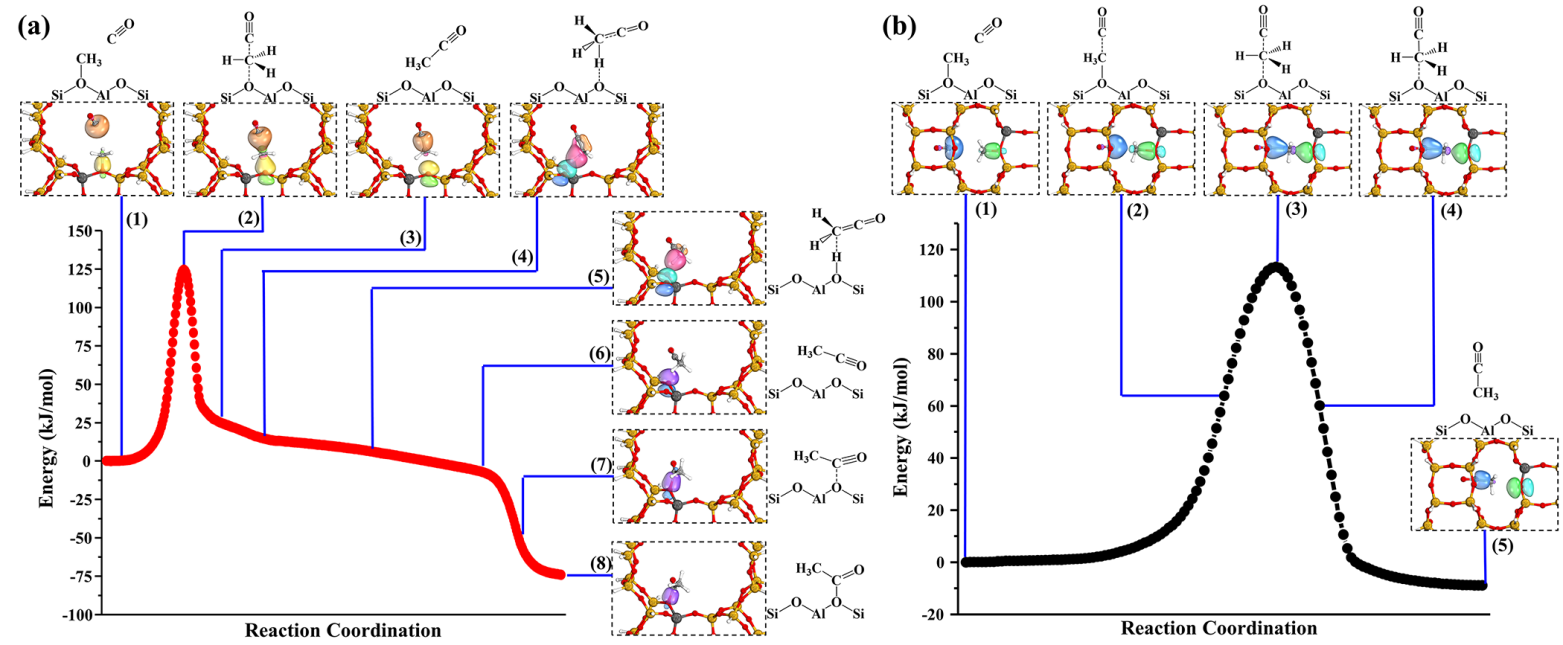
Potential energy surfaces and intrinsic bond orbitals of the C–C bond coupling between SMS and CO in (a) 12MR and (b) 8MR channels of MOR along with the intrinsic reaction coordinate based on model B shown in Figure S1. Note that orange balls of snapshots 1–3 in panel a and blue balls of snapshots 1–5 in panel b are the sp-hybrid lone pair (1-center orbital) of C≡O, and yellow balls in panel a and green balls in panel b are the C–O bond σ orbitals in zeolite framework AlO_4_^–^ tetrahedron; red balls and light blue balls of snapshots 4 and 5 in panel a are the C–H bond σ orbitals and p lone pairs in framework AlO_4_^–^, whereas the purple balls of snapshots 6–8 are the p lone pairs in AlO_4_^–^.

As mentioned above, unlike static DFT calculations, which are performed at 0 K, AIMD simulations are conducted under real experimental temperature and conditions. As such, besides surface acetyl, the acylium ion was also identified as a metastable intermediate in MOR-8MR during the carbonylation reaction because of the pore confinement effect. In this context, the formation of ketene intermediate and its role during the reaction process have been completely ignored in static DFT calculations. To address this conflict between static and dynamic calculations, the strictly static calculations on C–C bond formation were also conducted using intrinsic reaction coordinate (IRC) to verify the presences of various products in 8MR and 12MR channels of MOR. As shown in Figure 3, the potential energy surfaces (PES) and relevant intermediates and products so observed were all in good agreement with the minimum free energy paths (MFEP) depicted in Figure 1d obtained based on MTD-AIMD simulations. Thus, the obvious presence of acylium ion as a product in MOR-8MR was neglected by ignoring IRC calculations during static DFT calculations.

**Figure 4:**
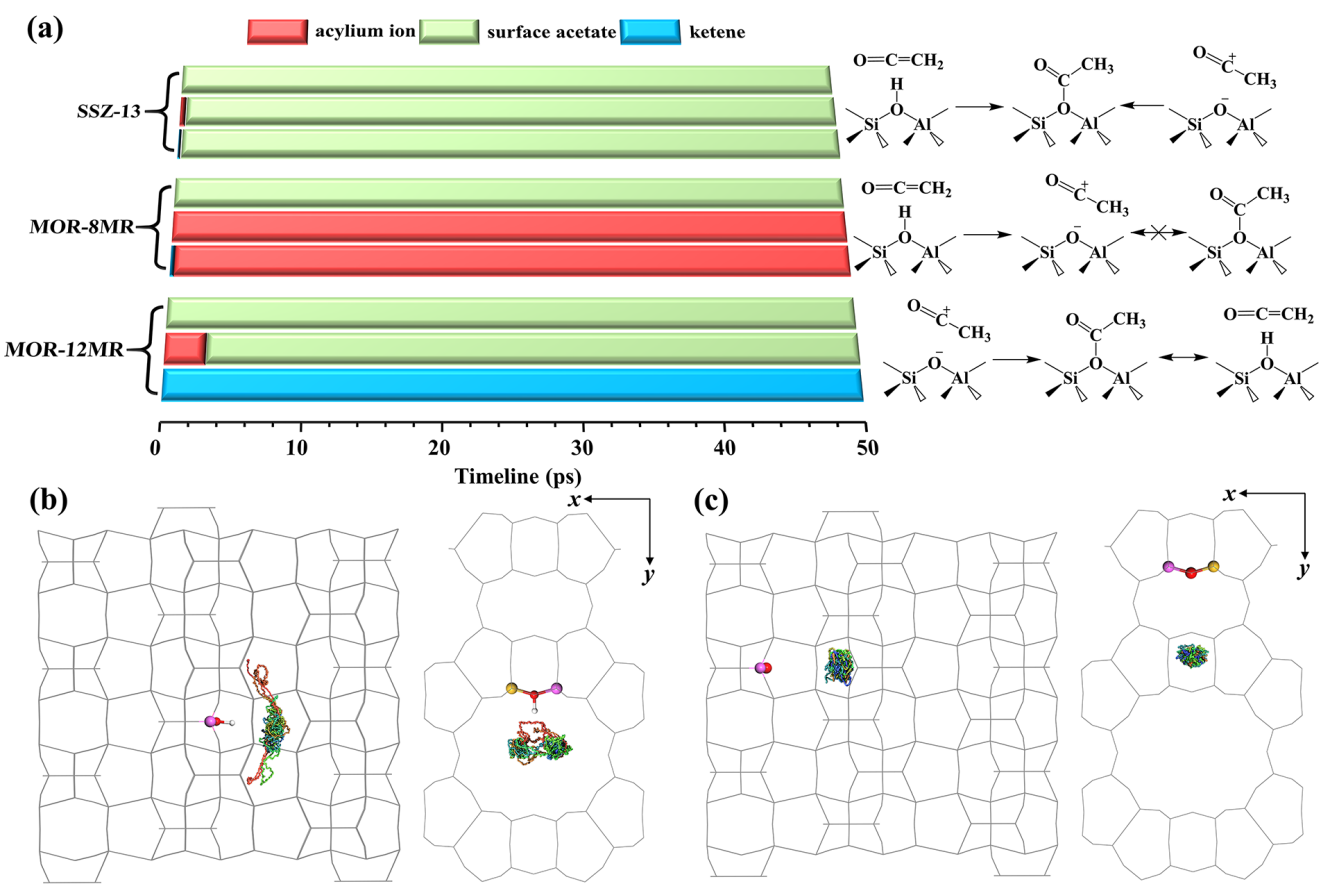
(a) Evolutions of acylium ion, surface acetyl, and ketene in SSZ-13, MOR-8MR, and MOR-12MR based on 50 ps AIMD simulations at 473 K. Side view and top view of trajectories for the COM (center of mass) of (b) CH_2_CO (ketene) in MOR-8MR and (c) CH_3_CO^+^ (acylium ion) in MOR-12MR during AIMD simulations.

**Figure 5:**
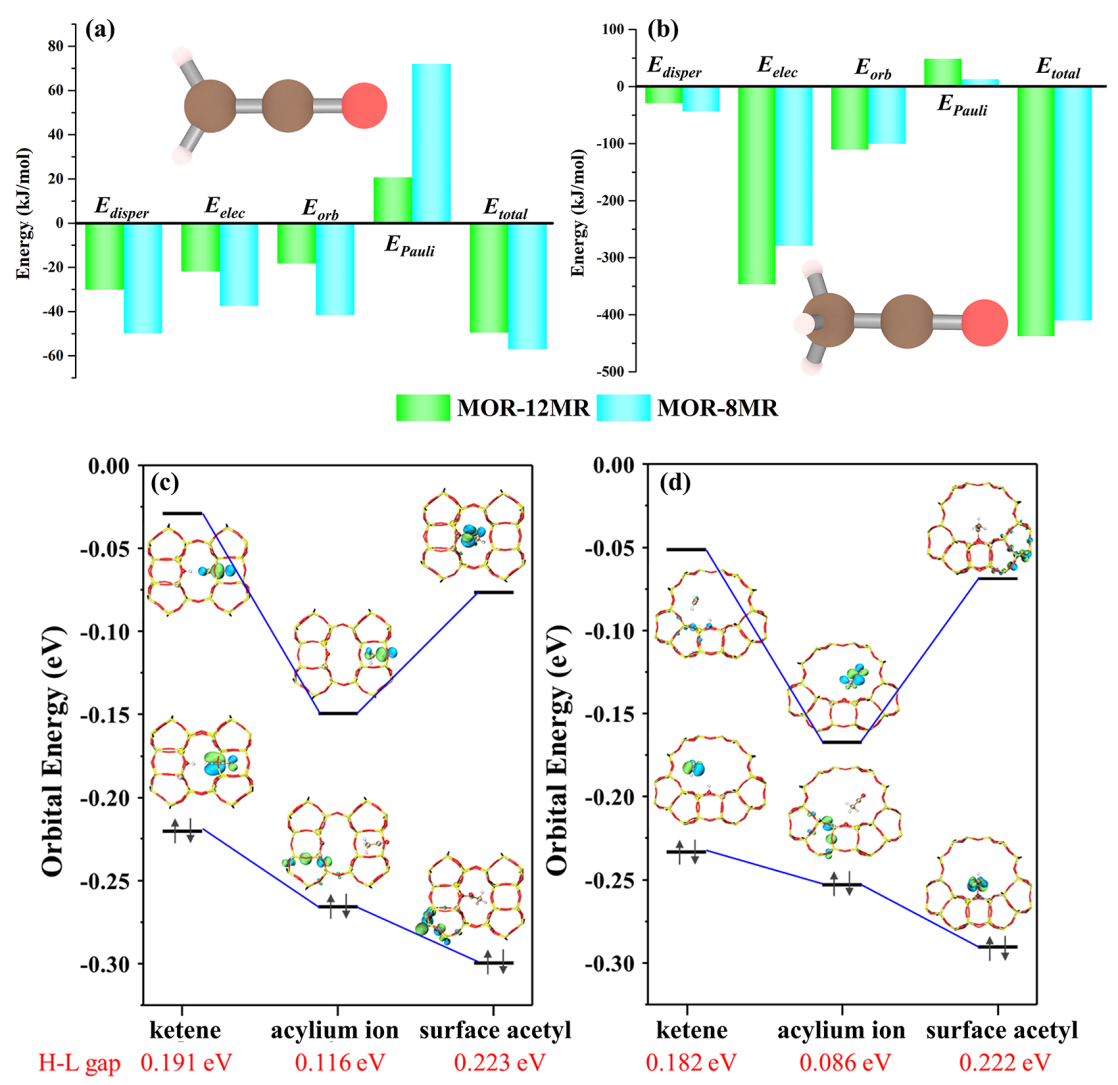
Energy decomposition analysis of host–guest interactions in MOR-8MR and MOR-12MR, guest species: (a) ketene and (b) acylium ion. Molecular frontier orbital energy and HOMO–LUMO (H-L) gap of ketene, acylium ion, and surface acetyl inside (c) MOR-8MR and (d) MOR-12MR.

Subsequently, a detailed intrinsic bond orbital (IBO) evolution was carried out to provide the evolution of localized molecular orbitals of bond formation/rupture processes along with IRC (see Movies S4and S5). After breaking the C–O σ orbital, the formation of C–C bond was provoked by the sp-hybridized lone pair (1-center orbital) of CO and the inversed sp^3^-hybridized orbital of SMS (see snapshots 1–3 in Figure 3a and 1–5 in Figure 3b) assigned to the typical S_N_2 mechanism. Unlike in the MOR-8MR, in which subsequently formed acylium ion (CH_3_CO^+^) was stabilized because of the confinement effect, it evolves further in MOR-12MR. As previously reported by Jensen and co-workers,^7^ our AIMD simulation results also revealed that evolution of CH_3_CO^+^ in MOR-12MR channels followed the sequence: first, it was deprotonated to form ketene (snapshot 4); next, the ketene orientation was adjusted to favor the formation of the C–O bond with shorter distance (snapshot 5)), and finally, protonation of ketene to form CH_3_CO^+^ again with the most suitable orientation and optimal C–O distance for the formation of surface acetyl (snapshots (6–8)). It should be noted that the formation of ketene did not occur solely via CH_3_CO^+^ protonation since the O–H σ bond orbital was not in its final form, but rather, merely through highly overlapping of C–H σ bond orbital and p lone pair of oxygen (snapshots (4–5)) in zeolite framework AlO_4_^–^ tetrahedron along with the IRC. Thus, it was evidenced that the static DFT calculations by ignoring the dynamic behaviors of intermediate species at real temperature led to misleading predictions of reaction intermediates and products, thus, inaccurate reaction pathways. In this context, AIMD simulation is far more superior in dealing with active intermediates during the reaction.

### 2.3 Stability, Activity, and Mobility of Ketene and Its Derivatives in Zeolites

The lifetime of intermediate is another key factor influencing the reactivity. To account for effects of conformational freedom, entropic effect, and temperature effect in a more realistic manner, we applied the regular 50 ps AIMD simulations at the NVT ensemble for SSZ-13, MOR-12MR, and MOR-8MR containing ketene, the acylium ion, and surface acetyl as intermediates. As illustrated in Figure 4a, ketene was found to be rapidly protonated by the proton in MOR-8MR to form an acylium ion. Unfortunately, no dynamic equilibrium between ketene and the acylium ion was detectable (see Figure 2b) because of the limited time scale. The acylium ion and surface acetyl, as the starting species, were found to remain unchanged throughout the 50 ps AIMD simulations, which indicates that the MOR-8MR have the stabilizing effect to these two species but none on ketene. Although surface acetyl was found to be strongly bonded to zeolite framework AlO_4_^–^ tetrahedron, its formation from acylium ion was hindered by two free energy barriers, as shown in Figure 1e. Thus, it is affirmative that the acylium ion is the candidate intermediate in MOR-8MR during carbonylation. In this context, the confined environment of MOR-8MR is responsible for the high stability and catalytic activity observed for the acylium ion. On the other hand, both ketene and surface acetyl were found to be quite stable in MOR-12MR throughout the 50 ps simulation period, whereas the acylium ion appears to transform quickly to surface acetyl because of strong electrostatic interaction. For SSZ-13, surface acetyl was found to be the only stable species during the reaction; both the acylium ion and ketene rapidly converted to surface acetyl. Thus, in terms of mobility of the stable species in zeolites, surface acetyl was found to bond tightly with framework AlO_4_^–^ in both MOR and SSZ-13.

As indicated by trajectories in Figure 4b, c, the acylium ion in MOR-8MR is found to move restrainedly under the confinement effect of MOR frameworks, whereas, in contrast, ketene has the higher mobility in MOR-12MR. In addition to the different confinement effects of MOR-12MR and MOR-8MR to ketene, the stronger acidic strength of BAS in MOR-8MR than that in MOR-12MR may be the other reason for the different protonation states of ketene.^43,44^ The diverse behaviors of ketene and acylium ion in MOR-12MR and MOR-8MR differentiates the activity of the various channels toward MA and AcOH formation. The long life of ketene and its high mobility in MOR-12MR would facilitate the migration of ketene from the 12MR channel to the 8MR channel via the 10MR window of the side pocket and then protonation to the acylium ion, which is located at the side pocket with low mobility as illustrated in Figure S3.

To render an in-depth understanding of the stabilities of the acylium ion and ketene in MOR, we exploited energy decomposition analysis (EDA) to explore their interactions with the zeolite framework. Relevant results are shown in Figure 5a, b and summarized in Table S3. The significantly higher value of *E*_Pauli_ observed for ketene (CH_2_CO) in MOR-8MR indicates the presence of strong Pauli repulsion toward ketene from the zeolite framework. Such mutual incompatibility between ketene and zeolite led to the short lifetime of ketene in MOR-8MR, as shown in Figure 4a. For the same reason, the stability of the acylium ion (CH_3_CO^+^) inside MOR-8MR should be attributed to the lower *E*_Pauli_ value. Moreover, *E*_elec_ is the primary energy component responsible for the stabilization of the acylium ion. Clearly, protonation of ketene to form the acylium ion in MOR-12MR led to a notable increase in *E*_Pauli_, in contrast to that observed in MOR-8MR. The above results clearly show that a good compatibility between MOR-12MR and ketene, as well as MOR-8MR and the acylium ion, can be inferred.

Furthermore, the reactivity of ketene, the acylium ion, and surface acetyl in MOR and SSZ-13 can also be validated by the HOMO–LUMO (H-L) gap as a correlation descriptor between the reactivity of species located in various confined voids.^45,46^ The molecular system with the lower H-L gap indicated that the molecular system can be easily excited with the higher reactivity. Relevant results are depicted in Figure 5c, d and Table S4. Note that the transformation process of ketene → acylium ion → surface acetyl is generally accompanied by the gradual decrease in HOMO energy in MOR. Moreover, the LUMO energy predicted for acylium ion is evidently lower than that for ketene and surface acetyl in zeolites. On the basis of the observed frontier orbital energies, it is obvious that the acylium ion possesses a higher reactivity than surface acetyl. Thus, in terms of the H-L gap energy, the trend of reactivity is as follows: surface acetyl < ketene < acylium ion. Moreover, as revealed by the AIMD simulations illustrated in Figure 4a, unlike MOR-12MR, the acylium ion was found to be stabilized in MOR-8MR at reaction temperature, as also confirmed by the lowest H-L gap (0.086 eV), hence the highest reactivity found for MA and AcOH formation. Therefore, as a derivative of ketene, the acylium ion in MOR-8MR is not only well stabilized but also exhibits a higher reactivity compared to ketene and surface acetyl, even in MOR-12MR and SSZ-13 zeolites.

On the basis of the stability, mobility, and activity of ketene, the acylium ion, and surface acetyl in MOR and SSZ-13, the geometry of internal voids ruled by the pore architecture are found to be responsible for the stabilization of the acylium ion during carbonylation reaction. Consequently, a swift reduction in the concentration of ketene should be an effective strategy for the prominent enhancement of catalytic performance. The presence of the larger amount of ketene facilitates its undesired dimerization and the formation of the precursor of carbonaceous deposits, and finally to catalyst deactivation.^13,14,47^ The superior stability of the acylium ion in MOR-8MR is therefore more beneficial in reducing the barrier for MA and AcOH formation by avoiding C–O bond cleavage in surface acetyl. Apparently, the MOR-8MR represent an ideal and active void for catalyzing the carbonylation reaction and, hence, for effective coupling of C–C bond and formation of MA and AcOH.

### 2.4 Reaction Intermediates Captured in MOR and SSZ-13 Zeolites

The above AIMD simulations demonstrate the unambiguous evolution of reaction intermediates in confined zeolites during the DME/methanol carbonylation. However, limited by the low sensitivity of spectrum characterization and the short life of reaction intermediates at the reaction temperature, it is hard to directly observe these intermediates at the reaction temperature of DME/methanol carbonylation. Acetyl chloride (CH_3_COCl), as the acylating agent, can be adsorbed on the BAS of H-MOR and further transformed into an acylium ion (eq 1), which was expected to remain unchanged or to be converted to ketene or surface acetyl (eq 2) in different channels of MOR as observed by AIMD simulations in Figure 4a.
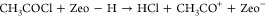
1
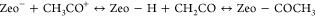
2Moreover, dechloridation of CH_3_COCl enables the capture of ketene and its derivatives solely in MOR by avoiding the other carbonylation species (methanol, CO, AcOH, MA, H_2_O, etc.). It also helps to reproduce the possible coke formation in the premise of abundant ketenes. Herein, SR-XRD, because of the benefits on the quality of data and the low temperature setup minimizing the thermal vibration contributions to anisotropic atomic displacement parameters, was employed to capture these reaction intermediates in MOR zeolites at 298 K. Subsequently, Fourier-transform infrared spectroscopy (FT-IR was used to follow the dynamic evolution of these intermediates with increasing temperature and different dosages of CH_3_COCl.

**Figure 6:**
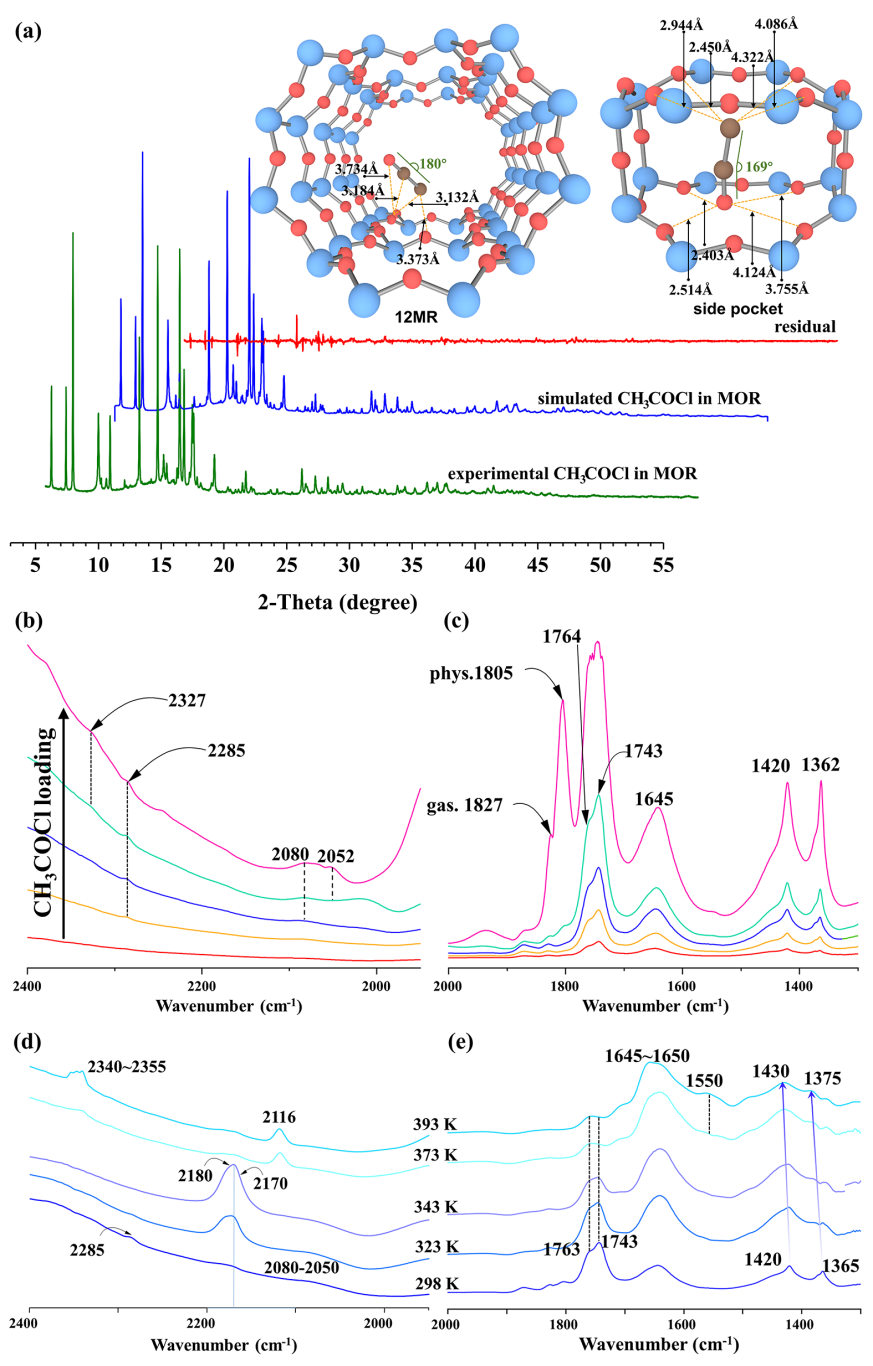
(a) Synchrotron X-ray powder diffraction (SR-XRD) and Rietveld refinement collected of dechlorinated CH_3_COCl in H-MOR zeolite (Si/Al = 10) at 298 K as compared by the simulated spectrum, as well as the geometrical parameters of host–guest interaction in 12MR channel and side pocket. FT-IR spectra of CH_3_COCl collected for (b, c) different loadings in H-MOR zeolite at (d, e) at different temperatures, both presented in (b, d) 2400–2000 cm^–1^ and (c, e) 2000–1300 cm^–1^ frequency regions. The intensity of spectra in H-MOR (c, e) was doubled to better visualize the observed effects.

Besides the MOR framework atoms, SR-XRD has captured the CCO fragments of dechlorinated CH_3_COCl in both the 12MR channel and the side pocket of H-MOR zeolite as displayed in Figure 6a and Figure S4. The detailed crystal parameters and structures of H-MOR and H-MOR adsorbed by the dechlorindated CH_3_COCl can be found in Tables S5–S7. The CCO fragments in the 12MR channel can be ascribed to ketene because the linear C–C–O angle represented the conjugated C=C and C=O double bonds in ketene, with high consistency to the AIMD simulations in Figure 4a. The CCO fragment in the side pocket (with the C–C–O angle of 169°) can be identified as the balance of two resonance structures of acylium ion (CH_3_–C≡O^+ ↔^CH_3_–C^+^=O) formed because of the nonuniform confinement effect of the side pocket and the flexibility of the C–C–O angle with single–double(or single–triple) bonds combination as well. (Figure 6a) Moreover, the acylium ion in the side pocket originates from the protonation of ketene instead of the dechloridation of CH_3_COCl, because the CH_3_COCl was prohibited from accessing the side pocket and the 8MR channel because of its kinetic diameter (Figure S5); the mobility of the ketene from the 12MR channel to the 8MR channel is both thermodynamically and kinetically favorable as displayed in Figure S3. As a validation of this point, in 50 ps AIMD simulations employed to sample the C–C–O angles of ketene and the acylium ion in MOR-8MR as displayed in Figure S6, the C–C–O angle of ketene is obviously distributed in a narrower range than that of the acylium ion, and the distribution of the C–C–O angle in the acylium ion is closer to 169° of SR-XRD. However, it is unreasonable to exclude the probability of ketene being in the side pocket of MOR, and the CCO fragments in the side pocket of MOR should be assigned to the averaged structures of the acylium ion and ketene. The nice consistency of the SR-XRD spectrum between the experiment and the simulation confirms the reliability of these assumptions. Interestingly, the surface acetyl species, found to be more stable than ketene and the acylium ion, were not detected by SR-XRD, most likely due to their irregular distribution in the MOR. The appearance of surface acetyl relies on the Al location and content (only 4.4 Al in each unit cell) in H-MOR (Si/Al = 10), and thus the disordered Al in the H-MOR zeolite finally led to the disappearance of surface acetyl in the crystal structure (see Figure 6a).

FT-IR spectra of different CH_3_COCl loadings adsorbed in H-MOR (Si/Al = 10) were collected at 298 K (Figure 6b, c and Figure S6a). The CH_3_COCl was dechlorinated to HCl and acetyl species what is manifested by the development of vibration–rotation bands of HCl (3100–2600 cm^–1^) and the band of the C=O stretching vibration of surface acetyl species (1645 cm^–1^), as reported by Cheng et al.^48^ The C=O bands at 1827, 1805, 1764, and 1743 cm^–1^ identify gas phase and various adsorbed states of CH_3_COCl (physical adsorption and hydrogen bond interaction with BAS), while the bands at 1420 and 1362 cm^–1^ originate from the bending vibration of the −CH_3_ groups. In contrast to H-MOR, the surface acetyl species were identified in H-SSZ-13 as the predominant species, as shown in Figures S7b and S8, and therefore surface acetyl moieties can be considered as the stable intermediates in H-SSZ-13 as consistent with AIMD simulations in Figure 4a. Moreover, some well-resolved bands in the 2400–2000 cm^–1^ region are detected as shown in Figure 6b. The 2327 and 2285 cm^–1^ signatures may be ascribed to acylium ion according to the experimental and calculated values of 2307 and 2304 cm^–1^, respectively, as reported by Mosley et al.^49^ These entities are possibly located at the side pocket, as illustrated in AIMD and SR-XRD. The bands located at 2080 and 2052 cm^–1^ are of frequency closer to 2152 cm^–1^ of ketene reported by Moore and Pimentel,^50^ and they may come from either ketene engaged in the strong interaction with HCl and unreacted CH_3_COCl or protonated ketene CH_2_=C=O^+^H.^49^ The analogous bands of 2070 and 2050 cm^–1^ were also detected in the spectrum of CH_3_COCl adsorbed in H-SSZ-13 (Si/Al = 11.5). (Figures S7b and S8) However, the unique band of acylium ion was observed exclusively in H-MOR confirming the specificity of MOR-8MR activity, as evidenced by this work and literature reports.^17,18,20−22^

The possible interconversion of intermediates and precursors of carbonaceous deposits was explored by FT-IR studies, the evolution of these three intermediates in the temperature range from 298 to 393 K was displayed in Figure 6d, e. The upshift of CH_2_ and CH_3_ vibrations from 1420 and 1365 cm^–1^ to 1430 and 1375 cm^–1^, respectively, implies that the rotations in C–H bonds are hampered. The alterations in CH_2_ and CH_3_ bands frequencies are accompanied by the appearance of new 1650–1555 cm^–1^ bands indicating that they originate from the same chemical moieties, i.e., surface acetyl species. When temperature increased to 323 K, the bands at 2285 and 2080–2050 cm^–1^ were consumed in favor of the bands at 2180 and 2170 cm^–1^. The latter bands can be representative of ketene^49^ located in 12MR channel no longer interacting with HCl or CH_3_COCl, as manifested by SR-XRD.(Figure 6a) Interestingly, the bands at 2170–2180 cm^–1^ are also eroded from the catalyst surface when the temperature further increased to 373 K being transformed to propyne (the C≡C stretching vibration band at 2116 cm^–1^) and CO_2_ (the bands at 2340–2355 cm^–1^).(Figure 6c and Table 1) Herein, propyne and CO_2_ could only have come from the dimerization of ketene and the further dissociation of diketene.(Figure S9) The formation of propyne and propadiene, as the precursor of carbonaceous deposits, could be one major reason for coke formation in DME carbonylation, and the presence of the lesser amount of ketene during the reaction should be more unfavorable for the formation of carbonaceous deposits. It is worth underlining that all three intermediates, i.e., surface acetyl, ketene, and acylium ion, were detected by SR-XRD and in FT-IR spectra as the products of CH_3_COCl dechloridation in H-MOR, but surface acetyl was the major species in H-SSZ-13. All these experimental observations are highly consistent with the conclusion about their stability in various zeolites obtained in the AIMD simulations.

**Table 1:** FT-IR Band Affiliations of C–O Bond Stretching Vibrations According to the Calculated Values and Referenced Values

			MOR zeolite	
species	ref	calcd^a^	gas^b^	ads^c^
surface acetyl	1630^48^			1645–1650
acylium ion	2307^49^	2304		2285, 2327
ketene	2152^49^	2189		2170–2180
CH_3_COCl	1818^51^	1915	1827, 1805	1743, 1763
CO_2_	2320–2375^52^	2388	2340–2355	
CH_3_C≡CH^d^	2160^52^	2169	2116	

a Calculated vibrational wavenumber by PBE-D3/6-311G(d, p) method.

b Species with weak interaction to MOR.

c Species were tightly adsorbed or confined by MOR.

d C≡C bond stretching vibration.

## 3 Conclusions

By invoking AIMD in conjunction with SR-XRD and FT-IR experiments, the roles of ketene and its derivates (viz., acylium ion and surface acetyl) associated with direct C–C bond coupling during DME/methanol carbonylation or MTO reaction over mordenite and SSZ-13 zeolites have been thoroughly investigated under realistic reaction conditions. It has been demonstrated that, although ketene is an indispensable intermediate during carbonylation reaction, the confinement effect exerted by the pore channels of zeolites also has a significant influence on the formation, stability, and transformation of ketene. Thus, the evolutions and roles of active and detrimental intermediates depend strongly on the structural framework and pore architecture of the zeolite catalysts. For 8MR channels of MOR, dispersion and electrostatic interactions exerted from the side pockets tend to provoke rapid protonation of ketene to form highly stable acylium ions exclusively, which are favorable toward high reactivity during the catalytic reaction. By contrast, in the 12MR channels of MOR a relatively long lifetime was observed for ketene, which tends to accelerate deactivation of zeolite (because of coking), and hence is detrimental to the reaction process as revealed by FT-IR investigations. Thus, we resolve, for the first time, a long-standing debate regarding the genuine role of ketene in zeolite catalysis. In addition, we demonstrated conclusively that, whether beneficial or detrimental, the presence of ketene is closely related to the structure and pore architecture of zeolites and thus is capable of providing important information for future development of superior/upgraded catalytic processes. As a paradigm, we have demonstrated that the confinement effect of zeolite, as well as details on the formation and transformation of active intermediates during the catalytic process, may readily be achieved by advanced *ab initio* molecular dynamic simulations.

## Supporting Information Available

Computational details for the AIMD simulations and experimental procedures for the SR-XRD and FT-IR (PDF)
Episode of AIMD simulation for the C–C bond formation process between SMS and CO in MOR-12MR (MP4)
Episode of AIMD simulation for the C–C bond formation process between SMS and CO in MOR-8MR (MP4)
Episode of AIMD simulation for the C–C bond formation process between SMS and CO in SSZ-13 (MP4)
Evolution of pivotal IBO during the C–C bond formation process between SMS and CO in MOR-12MR (MP4)
Evolution of pivotal IBO during the C–C bond formation process between SMS and CO in MOR-8MR (MP4)
