# Supplementary material for: Molecular Understanding of the Catalytic Consequence of Ketene Intermediates under Confinement
Source: J Am Chem Soc. 2021 Sep 3;143(37):15440–52. doi: 10.1021/jacs.1c08036 (PMC8461653; doi:10.1021/jacs.1c08036)
Supplement: Supplementary file 1 — ja1c08036_si_001.pdf [file ja1c08036_si_001.pdf]

Supporting Information

Molecular Understanding of the Catalytic Consequence of Ketene Intermediates under Confinement

Wei Chen,[a] Guangchao Li,[a,c,f] Xianfeng Yi,[a] Sarah J. Day,[d] Karolina A. Tarach,[b] Zhiqiang Liu,[a] Shang-Bin Liu,[e] Shik Chi Edman Tsang,[c, *] Kinga Góra-Marek,[b, *] and Anmin Zheng,[a, f, *]

[a]*State Key Laboratory of Magnetic Resonance and Atomic and Molecular Physics, National Center for Magnetic Resonance in Wuhan, Wuhan Institute of Physics and Mathematics, Innovation Academy for Precision Measurement Science and Technology, Chinese Academy of Sciences, Wuhan 430071, P. R. China.*
E-mail: zhenganm@wipm.ac.cn
[b]*Faculty of Chemistry, Jagiellonian University in Krakow, Gronostajowa 2, 30-387 Krakow, Poland.*
E-mail: kinga.gora-marek@uj.edu.pl
[c]*Wolfson Catalysis Centre, Department of Chemistry, University of Oxford, Oxford OX1 3QR, United Kingdom.*
E-mail: edman.tsang@chem.ox.ac.uk
[d]*Diamond Light Source Ltd., Harwell Science and Innovation Campus, Didcot, OX11 0DE, U.K*
[e]*Institute of Atomic and Molecular Sciences, Academia Sinica, Taipei 10617, Taiwan.*
[f]*University of Chinese Academy of Sciences, Beijing 100049, P. R. China.*

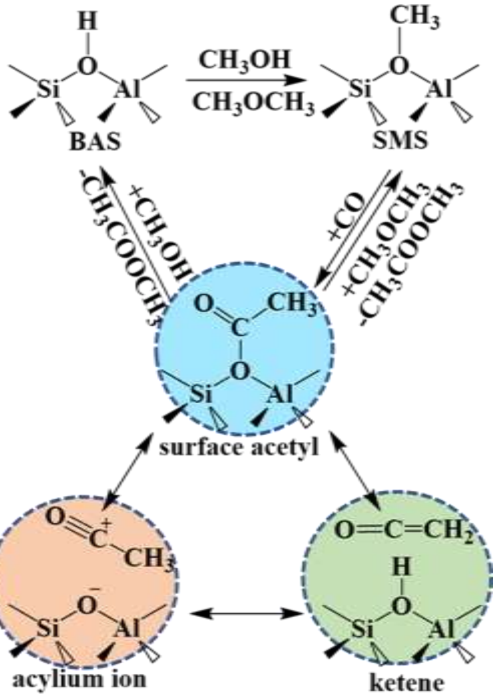

Scheme S1. General mechanism of zeolites catalyzed carbonylation.

1. Computational Details

1.1 Periodic DFT Caculations

The topologies of mordenite (MOR) were extracted from the official website of the International Zeolite Association (IZA).[S1] The cell parameters of SSZ-13 and MOR zeolites are ($a = b = 13.67$ Å, $c = 14.77$ Å) and ($a = 18.26$ Å, $b = 20.53$ Å, $c = 15.08$ Å), respectively. For the model of SSZ-13, one of silicon or phosphorus in T1-O2-T1 sites was replaced by aluminum or silicon to introduce the surface methoxy species (SMS) on O2, as shown in **Figure S1**. On the other hand, one of silicon in T4-O10-T4 (12MR) and T3-O8-T3 (8MR) were replaced by aluminum to introduce the SMS for MOR, and denoted as MOR-12MR and MOR-8MR, respectively. To account for zeolite framework flexibility and guest molecule dynamics in full at designated reaction temperatures, *ab initio* molecular dynamic (AIMD) simulations were performed by periodic density functional theory (DFT). This is carried out by first performing equilibrium AIMD simulations of reactant state species for 5 *ps* at the isothermal-isobaric (NPT) ensemble, in which the amount of substance (N), pressure (P) and temperature (T) are conserved, to relax the cell parameters and atom positions.

Next, the metadynamics (MTD) method was exploited to explore the occurrence of carbonylation in MOR-12MR as well as MOR-8MR during at least 35 *ps* AIMD simulations. Finally, the thermal stability of various intermediates (*viz.*, CH_2CO, Zeo-$OCOCH_3$, and CH_3CO^+) were sampled during 50 *ps* canonical (NVT, *i.e.*, moles, volume, and temperature) AIMD simulations. Notably, the free energy profile in **Figure 1c** and the critical distance evolutions of direct C-C bond coupling in **Figure 2e** were reproduced based on our pervious work.[S2]

All AIMD simulations were employed by CP2K software,[S3] and the linked PLUMED code[S4] was used to carry out the MTD simulations. The Perdew–Burke-Ernzerhof (PBE) functional[S5] with consideration of Grimme D3 dispersion corrections,[S6] *i.e.*, the PBE-D3 functional, was chosen for the DFT calculations. Limited by large computational cost of triple-zeta basis set in AIMD simulations, the double-zeta (ζ) valence polarized (DZVP) basis set[S7] together with the Goedecker-Teter-Hutter (GTH) pseudopotential[S8] were used for the system. We have tested the convergence of DZVP basis set with SZV, TZVP, and TZVPP basis sets by the intrinsic activation energy ($\Delta E^‡$, 0 K) of C-C bond coupling of SMS and CO in MOR-12MR, MOR-8MR and SSZ-13 as listed in **Table S1**. Compared to the qualitative error on $\Delta E^‡$ by single-zeta basis set (SZV), double-zeta plus polarized basis set (DZVP) can quantificationally and quantitatively describe the $\Delta E^‡$ with the error smaller than 11 kJ/mol relative to triple-zeta plus polarized basis set (TZVP) and double polarized basis set (TZVPP). Notably, PBE-D3 functional used in this work, usually underestimates the free energy barriers in zeolite chemistry according previous benchmark studies,[S9, S10] our static DFT calculations based on cluster model also confirmed this point by the free energy barriers of PBE-D3/dgdzvp and B3LYP-D3/dgdzvp in **Table S2**. However, the static periodic calculations and MTD-AIMD simulations based on PBE-D3/DZVP carried out the free energy barriers highly closed to that by the hybrid functional of HSE06, B3LYP and *w*B97XD.[S11, S12] Therefore, the PBE-D3/DZVP method used here is reasonable to qualitatively and quantitatively compare the barriers of C-C bond formation between SMS and CO in different zeolites. During the self-consistent field (SCF) procedure, a 360 Ry density CUTOFF criterion with finest grid level was employed along with multi-grids number 4 (NGRID 4 and REL CUTOFF 70). The temperature was controlled by a chain of five Nosé-Hoover thermostats,[S13] and the integration time step was set to 0.5 *fs* during the AIMD simulations.

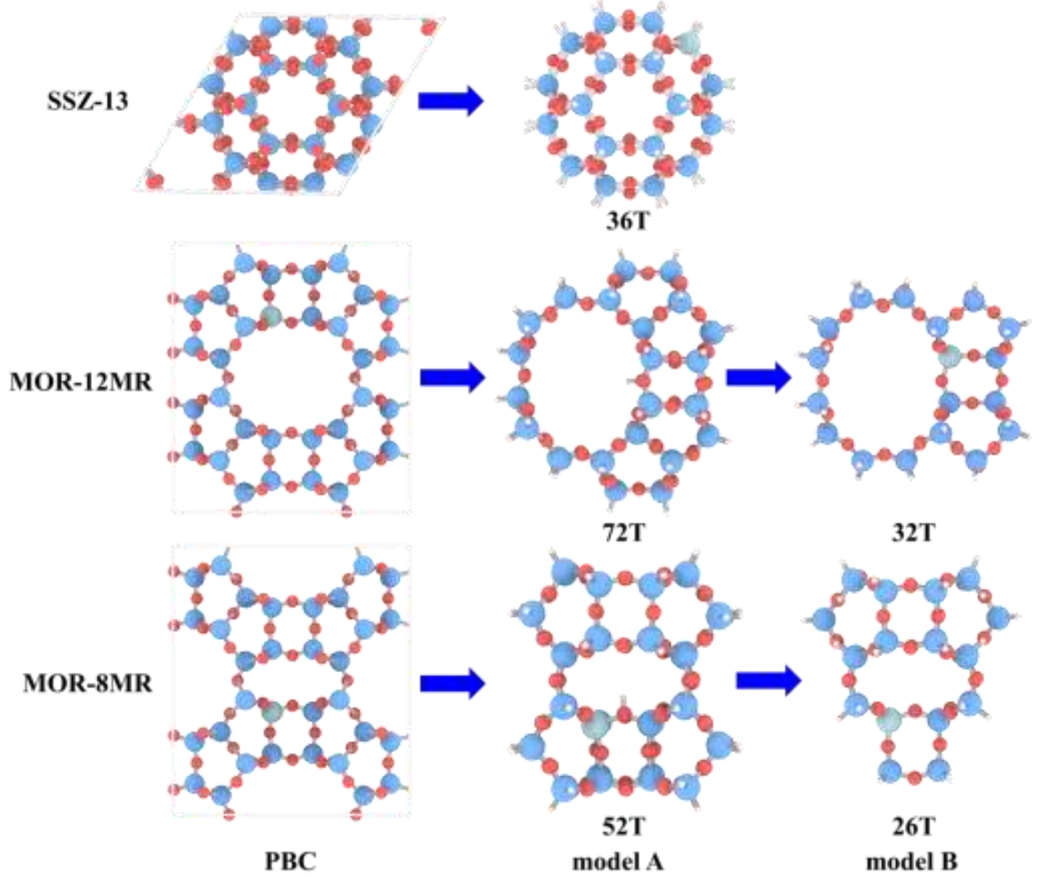

Figure S1. Periodic models and cluster models of CHA and MOR zeolites used under periodic boundary conditions (PBC) during the AIMD simulations. Cluster models A were used for energy decomposition analysis (EDA) and prediction of orbital energies, while models B were adopted for the intrinsic bond orbital (IBO) analyses with intrinsic reaction coordinate (IRC) pathways.

Metadynamics (MTD) simulation[S14] was employed to explore the C−C bond coupling between SMS and carbon monoxide (CO). This is done by first optimizing the geometrical structures and cell parameters of the configuration by periodic DFT followed by a 5 *p*s NPT molecular dynamics simulation to relax the structure and cell parameters under experimental conditions such as temperature and pressure. Accordingly, MTD simulations were performed at 473 K in the NVT ensemble; an advanced sampling technique employed for enhancing the probability of sampling chemical reactions or rare events provided that a limited number of collective variables (CV) describing the reaction coordinates were well-defined. Nonetheless, MTD simulation is normally biased by regularly spawning Gaussian hills along the chosen CVs, which was defined by coordination numbers (CN):

$$\mathrm{CN} = \sum_{ij} \frac{1 - \left(\frac{r_{ij} - d_0}{r_0}\right)^n}{1 - \left(\frac{r_{ij} - d_0}{r_0}\right)^m}$$

where r_{ij} represents the interatomic distance between atoms i and j and r_0 is the reference distance. In this work, parameters d_0, n, and m were set to 0, 6, and 12, respectively. Accordingly, CVs may be defined in terms of CNs, *viz.*, CV1 = CN(C_{SMS}–O_{Zeo}); CV2 = CN(C_{SMS}–C_{CO}), which were used to describe the proton transfer process, as shown in **Scheme S2**. By setting a reference distance of 2.1 Å and 1.5 Å for CN(C_{SMS}–O_{Zeo}) and CN(C_{SMS}–C_{CO}), respectively, and a Gaussian hill width and height of 0.035 and 2 kJ/mol for both CV1 and CV2, and a spawning every 50 *fs*, MTD simulations were allowed to continue till the height of additional hills no longer affect the resultant free energy profile. Based on the sum of spawned Gaussian hills, the 2D Gibbs free energy profile of the reaction may be reconstructed. Accordingly, a lowest free energy path (LFEP) was derived by means of the MEPSA software.[S15]

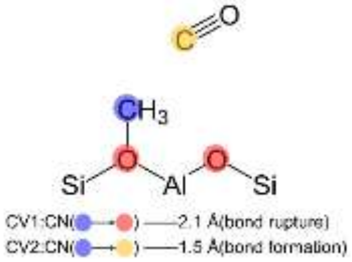

Scheme S2. Assignments of collective variables (CV1 and CV2) associated with the C–C bond coupling between SMS and CO during MTD simulations.

Umbrella sampling method, as the other free energy sampling techiques, was employed to explore the mobility of ketene and acylium ion in MOR. To describe the diffusion path of ketene and acylium ion between 8MR and 12MR channels, we define the distance (***d***) between two center of mass (COM) as the collective variable (CV), one COM is the ketene or acylium, the other is the 8MR window of side pocket oppsite to BAS, eight windows are respectively the independent simulation that represents a point along the CV from 7 to 0. The additional potential introduced by a simple harmonic term:

$$U(s) = \frac{k}{2}(x - x_0)^2$$

Where x is the real-time value of CV, x_0 is the value of specificed value of CV, k is set to 30 kJ/mol for ketene from 12MR to 8MR channel. All biased umbrella sampling simulations were consisted 10^4 steps at NVT ensemble, and the setting to k lead to the reasonable sampling indicated by the inter-overlaping of different windows. The free energy profile was generated by the sampling obtained in each window based on the weighted histogram anaylsis method.[S16-S17]

1.2 Cluster Model Calculations

The intrinsic bond orbitals (IBO) are a non-empirical form of localized molecular orbitals that furnish quantitative interpretation of bonding by assigning electrons in a doubly occupied IBO to the individual atoms.[S18] Together with the intrinsic reaction coordinate (IRC) pathways, IBO evolutions are capable of providing detailed information of orbitals and electrons during bond formation/rupture to ascertain the reaction mechanism. The IBO were obtained with the IboView program[S19] using the B3LYP-D3 and PBE-D3 methods with the 6-31G(d, p) basis set, whilst the IRC pathways were calculated by using the Gaussian 09 program.[S20] To circumvent the computational cost, relevant IBO and IRC calculations were conducted based on simplified cluster **model B** (*cf.* **Figure S1**).

Energy decomposition analysis (EDA) was used to better understand the confinement effect of intermediates in different channels based on static cluster calculations. Based on cluster models clipped from the optimized periodic structures (*i.e.*, **model A**; see **Figure S1**), detailed and precise analyses of host-guest and guest-guest interactions may be accomplished based on the ETS-NOCV EDA approach,[S21] which combined the extended transition state (ETS) method with the natural orbital for chemical valence (NOCV) theory by means of the Amsterdam density functional (ADF) program.[S22, S23] The PBE-D3 functional was chosen for the DFT calculations and EDA to warrant the uniformity between dynamic and static calculations. By invoking the frozen core approximation,[S24] a triple-zeta (TZ) Slater type orbital (STO) basis set containing two polarization functions (namely, TZ2P) was adopted for all elements to describe interactions between intermediates and the zeolite framework. Auxiliary STO functions, centered on all nuclei, were used to fit the electron density and to obtain accurate Coulomb potentials in each SCF cycle. For the ETS-NOCV EDA scheme, the interaction energy (ΔE_{int}) between the fragments may further be divided into four components:

$$\Delta E_{\text{int}} = \Delta E_{\text{Pauli}} + \Delta E_{\text{elec}} + \Delta E_{\text{orb}} + \Delta E_{\text{disper}}.$$

Where the four energy terms respectively account for the Pauli repulsive interaction among occupied orbitals on the fragments (ΔE_{Pauli}), the classical electrostatic interaction between two fragments (ΔE_{elec}), electron distribution of the constituent molecules (ΔE_{orb}), the dispersion interaction (ΔE_{disper}) due to the use of dispersion corrected PBE-D3 functional.

2. Experimental Methods

2.1 Sample preparation

Before adsorption of acetyl chloride, H-MOR (Si/Al=10) was shaped in the form of thin wafer (ca. 8.5 mg/cm^2) and place in quartz IR cell connected to a high vacuum line for dehydration. The temperature was gradually increased at a rate of 1K/min and the sample was kept at a final temperature of 673 K at a pressure below 10^{-3} Pa overnight. After the samples cooling down to ambient temperature, a known amount of acetyl chloride was introduced into the sample, and then the sample glass tube was sealed.

2.2 Synchrotron radiation X-ray diffraction

High-resolution SXRD data were collected at beamline I11, Diamond Light Source, Harwell, UK. The energy of the incident X-ray was set at 15 keV. The wavelength (l=0.826576(10) Å) and the 2θ zero point (ZP=0.000361(2)°) were determined by fitting the diffraction data of high-quality silicon powder (SRM640c). Before data collection, sample was loaded into borosilicate glass capillaries (0.5 mm ID) in a glove box. Glass wool was packed on top of the sample. The SR-XRD data were collected in a Debye-Scherrer geometry using multi-analyzer crystals (MAC) detectors in the 2θ range of 0–150° with 0.001° data binning. Each data set was collected for 1 h for good statistics.

2.3 FT-IR studies

Before adsorption of acetyl chloride, H-MOR (Si/Al=10) was shaped in the form of thin wafer (6 $mg\cdot cm^{-2}$) and place in quartz FT-IR cell connected to a high vacuum line. The sample was *in situ* thermally treated at 623K under high vacuum ($p = 10^{-5}$ mbar) for 1 h, then cooled down to room temperature (250K). The known doses of acetyl chloride (Sigma Aldrich) was adsorbed on the sample which has been tracking by the recording the spectra. The sorption of acetyl chloride was performed till the total or half consumption of the Si(OH)Al band. The FT-IR experiments were performed in rapid-scan mode (80 kHz) using Vertex 70 spectrometer (Bruker). Each spectrum (5

scans) was aquired within 1 sec with the spectral resolution was 2 cm^{-1}.

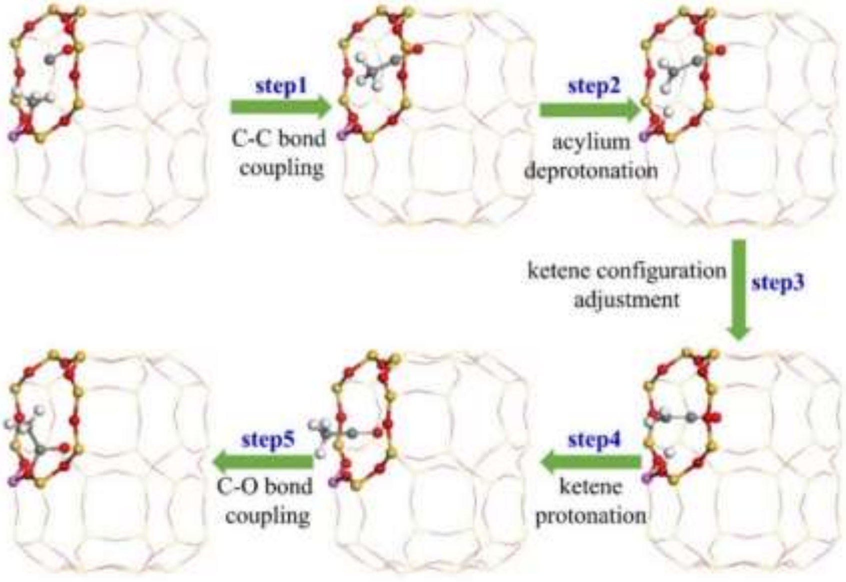

Figure S2. Proposed evolution of ketene and its role in the formation of surface acetate in SSZ-13.

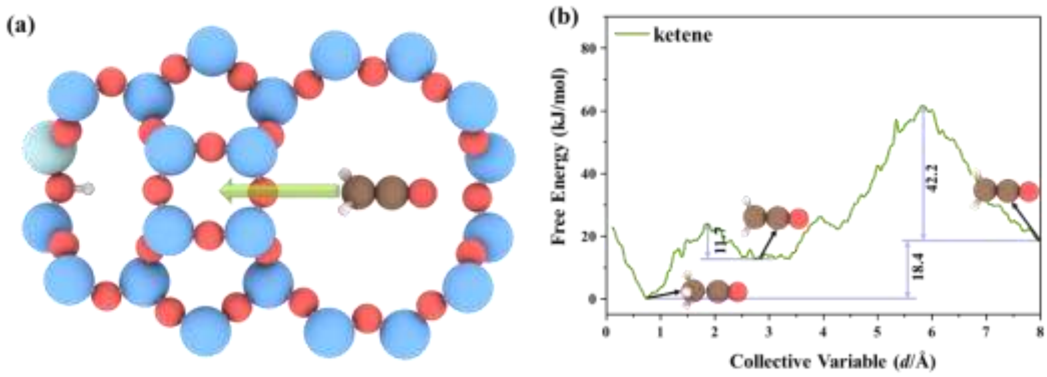

Figure S3. Schematic diagram for: (a) ketene diffusion from 12MR channel to 8MR channel, (b) ketene along with the COM distance between ketene and 8MR window (close to BAS) at 473 K.

To explore the mobility of neutral ketene from 12MR channel to 8MR channel in MOR, the diffusion behaviors of ketene in H-MOR have been carried out by umbrella sampling method during the AIMD simulations as displayed in **Figure S5a** and **S5b**, (see Computational Details and sampling region of **Figure S4** in Supporting Information). The diffusion process of ketene from 12MR channel to 8MR channel is both thermodynamically and kinetically favorable by overcoming a smaller free energy span of 42.2 kJ/mol with 18.4 kJ/mol decreasing on free energy, because ketene has been protonated to be acylium ion with a small free energy span of 11.7 kJ/mol in the path to gradually approach BAS at $d = 1.9$ Å. It's confirmable that ketene in MOR-12MR can spontaneously diffuse into the MOR-8MR and protonate to be acylium ion at reaction temperature.

Table S1. Basis set tests for the intrinsic activation energy ($\Delta E^{\ddagger}$,0 K) of C-C bond coupling of SMS and CO in MOR-12MR, MOR-8MR and SSZ-13.

Basis set	MOR-12MR	MOR-8MR	SSZ-13
SZV	119.1	100.2	141.2
DZVP	97.5	73.7	88.9
TZVP	85.3	72.8	92.0
TZVPP	93.7	84.6	100.0

Table S2. Comparison on free energy barriers ($\Delta G^{\ddagger}$) of carbonylation between SMS and CO in MOR zeolites, the increasing free energy of CO adsorption has been considered into $\Delta G^{\ddagger}$.

Methods	Model	T(K)	8MR	12MR	SSZ-13	References
MTD-AIMD, PBE-D3/DZVP	periodic	473K	128.6	155.7	143.0	This work
Static, PBE-D3/DZVP	periodic	473K	131.3	154.9	139.8	This work
Static, PBE-D3/TZVP	periodic	473K	137.5	147.1	149.7	This work
Static, PBE-D3/TZVPP	periodic	473K	117.8	158.0	145.5	This work
Static, PBE-D3/dgdzvp	cluster	473K	110.5	133.2	136.6	This work
Static, B3LYP-D3/dgdzvp	cluster	473K	126.6	156.8	162.0	This work
Blue moon-AIMD, PBE-D2/PAW	periodic	440K	102.4	129.6	-	ref [S11]
MLPT,[a] PBE	periodic	440K	110.2	118.6	-	ref [S11]
MLPT,[a] PBE-MBD	periodic	440K	105.1	129.4	-	ref [S11]
MLPT,[a] vdw-DF2-B86R	periodic	440K	107.7	139.7	-	ref [S11]
MLPT,[a] HSE06	periodic	440K	136.3	142.4	-	ref [S11]
Static, PBE-D2/PAW	periodic	440K	99.8	115.5	-	ref [S11]
Static, *w*B97XD/6-31G(d,p)	cluster	423K	123.2	158.8	-	ref [S12]
Static, BEEF-vdW/PAW	periodic	438K	175.6	185.6	-	ref [S25]
Static, B97D/6-31G(d,p)/6-311G(d,p)	cluster	0K	54.0	80.3	-	ref [S26]
Static, B3PW91/6-31G(d,p)/6-311G(d,p)	cluster	0K	98.3	107.1	-	ref [S27]

[a]machine learning thermodynamic perturbation theory.

Table S3. Energy decomposition analysis of interactions between ketene (CH_2CO) and H-Zeo, and acylium ion (CH_3CO^+) and Zeo^- in MOR-12MR, MOR-8MR and SSZ-13.

Energy terms	Definition	MOR-12MR		MOR-8MR		SSZ-13	
		CH_2CO	CH_3CO^+	CH_2CO	CH_3CO^+	CH_2CO	CH_3CO^+
E_{disper}	Dispersion interaction	−30.08 (42.9%)	−29.12 (6.0%)	−49.88 (38.7%)	−43.48 (10.3%)	−32.01 (39.7%)	−35.34 (6.0%)
E_{elec}	Electrostatic interaction	−21.83 (31.1%)	−346.65 (71.3%)	−37.5 (29.1%)	−279.00 (66.0%)	−24.88 (30.8%)	−420.62 (71.9%)
E_{orb}	Orbital distribution	−18.27 (26.0%)	−110.28 (22.7%)	−41.57 (32.2%)	−100.43 (23.7%)	−23.84 (29.5%)	−128.79 (22.0%)
E_{Pauli}	Pauli repulsive interaction	20.64	48.66	71.96	12.78	19.84	69.73
E_{total}	Total interaction energy	−49.54	−437.39	−56.99	−410.13	−60.89	−515.02

Table S4. Orbital energies and HOMO-LUMO (H-L) gaps (in eV) of ketene, acylium ion, and surface acetate in MOR-8MR, MOR-12MR and SSZ-13.

Zeolite	Intermediate	HOMO	LUMO	H-L gap
MOR-8MR	Ketene	–0.233	–0.051	0.182
	Acylium ion	–0.253	–0.167	0.086
	Surface acetyl	–0.291	–0.069	0.226
MOR-12MR	Ketene	–0.220	–0.029	0.191
	Acylium ion	–0.266	–0.150	0.116
	Surface acetyl	–0.300	–0.077	0.223
SSZ-13	Ketene	–0.228	–0.026	0.202
	Acylium ion	–0.236	–0.124	0.113
	Surface acetate	–0.251	–0.090	0.161

Table S5. Crystallographic data and details of pristine H-MOR (Si/Al=10), acetyl/H-MOR (Si/Al=10) based on SR-XRD.

Samples	pristine H-MOR	acetyl/H-MOR
Crystal system	Orthorhombic	Orthorhombic
Space group	Cmcm	Cmcm
2θ range refinement	3 – 55 º	3 – 55 º
Detector	Multi-analyser crystals	Multi-analyser crystals
Refinement methods	Rietveld	Rietveld
a (Å)	18.09 (5)	18.17 (2)
b (Å)	20.25 (6)	20.33 (2)
c (Å)	7.46 (9)	7.48 (5)
V ($Å^3$)	2737.93 (33)	2765.96 (64)
Rwp / Rp / Rexp (%)	6.4/4.40.79	5.7/4.2/1.4
Wavelength (Å)	0.826576	0.826576
2θ Zero point (°)	-0.000361	0.000361
Gof $\chi 2$	8.0	4.02

Table S6. Crystallographic information files from the Rietveld refinement of pristine H-MOR (Si/Al=10) based on SR-XRD.

Species	Atom	X	Y	z	SOF	Beq (Å^2)
Zeolite framework	O1	0.27765`	0.00000`	0.00000	1	4.82182
	O2	0.33378`	0.08193`	0.21845	1	17.5263
	O3	0.37546`	0.08846`	0.94172	1	0.00000
	O4	0.23929`	0.12188`	1.00705	1	0.61974
	O5	0.34280`	0.30830`	0.15075	1	20.0000
	O6	0.25	0.25	0.00283	1	12.6368
	O7	0.36801	0.31269`	0.93048	1	0.97479
	O8	0.00000	0.41293`	0.2500	1	12.3604
	O9	0.09712	0.30707	0.2500	1	2.49743
	O10	0.0000	0.19268	0.2500	1	0.00003
	Si1	0.30674	0.07539	0.03272	1	0.09917
	Si2	0.30117	0.31541	0.03804	1	0.09917
	Si3	0.09313	0.38177	0.2500	1	0.09917
	Si4	0.08471	0.22516	0.2500	1	0.09917

Table S7. Crystallographic information files from the Rietveld refinement of CH_3COCl/H-MOR (Si/Al=10) based on SR-XRD.

Species	Atom	X	Y	z	SOF	Beq (Å^2)
Zeolite framework	O1	0.27662	0.0000	0.0000	1	0.00000
	O2	0.33607	0.08025	0.2500	1	4.22735
	O3	0.37583	0.08613	0.93274	1	2.04639
	O4	0.23359	0.12584	1.00041	1	1.63773
	O5	0.33511	0.30758	0.2500	1	2.93427
	O6	0.2500	0.2500	0.0000	1	0.00000
	O7	0.36771	0.31241	0.94338	1	3.34273
	O8	0.00000	0.41129	0.2500	1	0.00000
	O9	0.08839	0.29909	0.2500	1	1.77262
	O10	0.0000	0.19268	0.2500	1	0.00000
	Si1	0.30066	0.07358	0.03254	1	0.76537
	Si2	0.30365	0.30919	0.04476	1	0.76537
	Si3	0.08803	0.38425	0.2500	1	0.76537
	Si4	0.08383	0.22341	0.2500	1	0.76537
CCO in 12MR	C2	-0.05752	-0.04625	0.53268	0.6104	19.915
	C1	-0.01583	-0.09033	0.39742	0.6104	19.915
	O-3	-0.09141	-0.01042	0.64263	0.6104	19.915
CCO in side pocket	C2a	0.04211	0.32830	0.69027	0.45753	19.915
	C1a	0.04909	0.25368	0.72914	0.45753	19.915
	O-3a	0.03845	0.39018	0.68841	0.45753	19.915

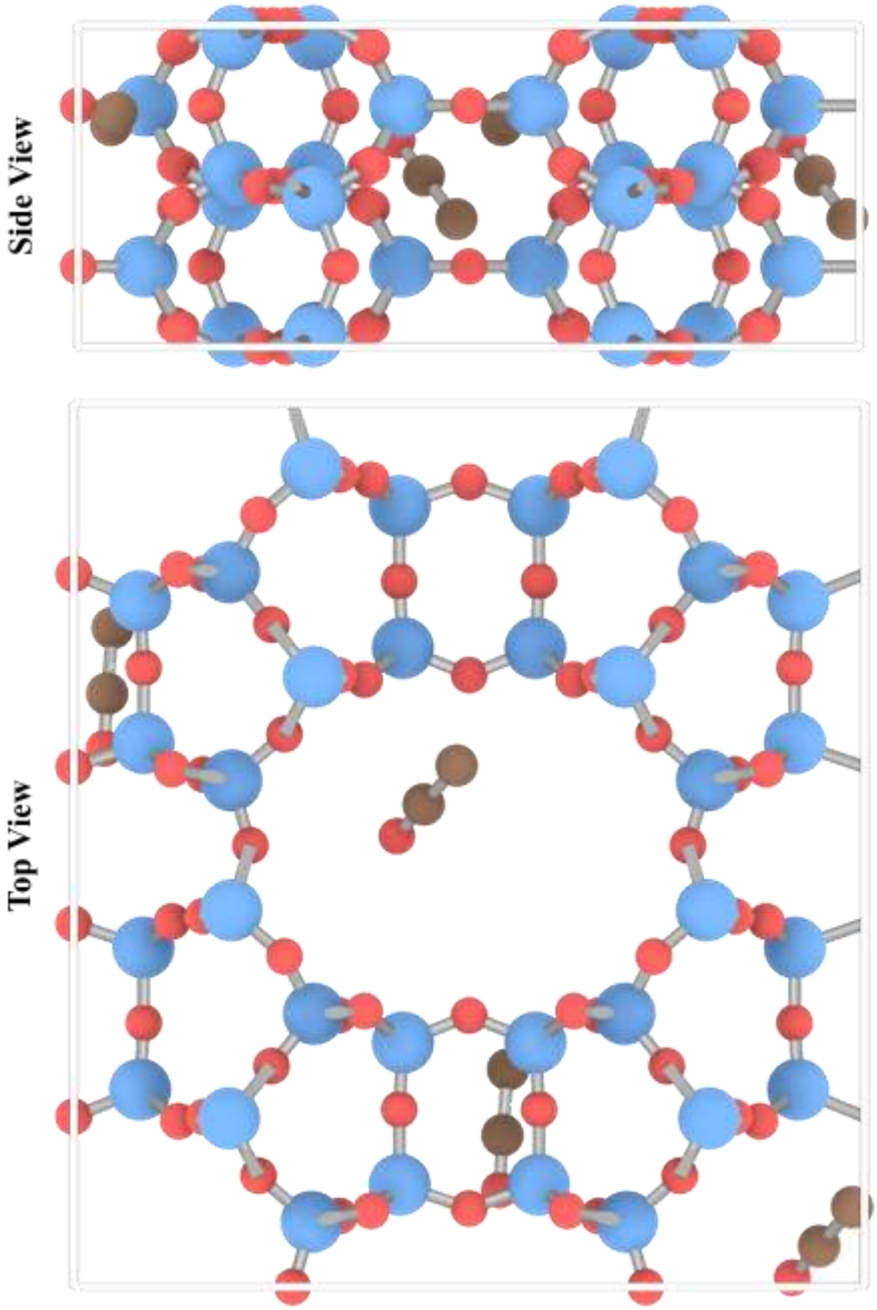

Figure S4. Top and side views of CH_3COCl/H-MOR unit cell; blue: silicon, red: oxygen, black: carbon.

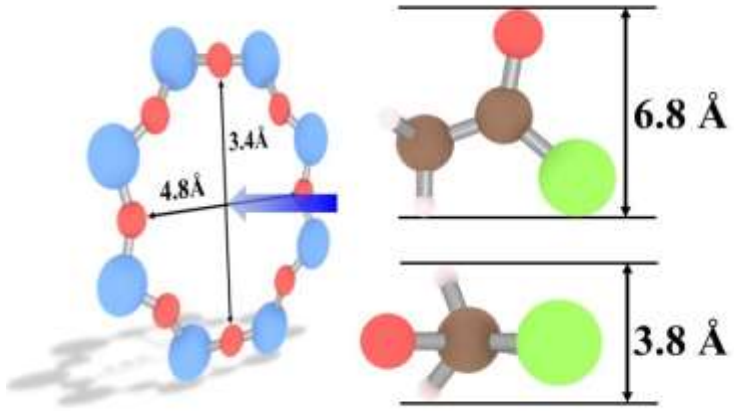

Figure S5. 8MR window size of side pocket in MOR and kinetic diameters of CH_3COCl, CH_2CO and CH_3CO^+. The window size obtained from IZA website, and the kinetic diameter of CH_3COCl are calculated according the method by Mehio *et. al.*[S28] but the kinetic diameters of CH_2CO and CH_3CO^+ are orginated from these of ethene and methane.

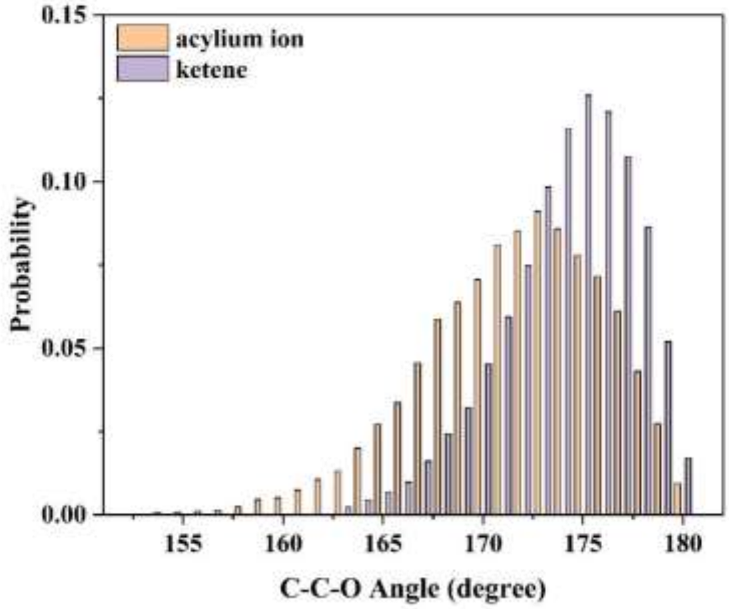

Figure S6. The C-C-O angle distributions in acylium ion and ketene species located in MOR-8MR obtained form the 50 ps AIMD simulation at 293K.

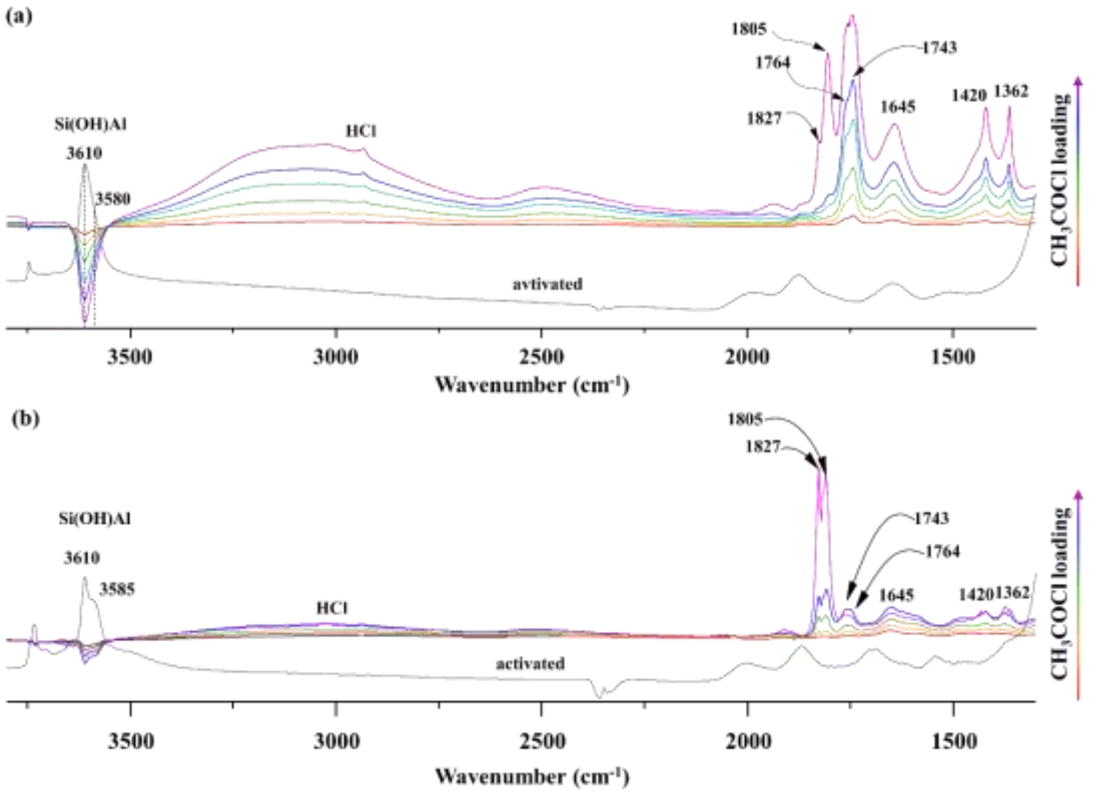

Figure S7. The FT-IR spectra in 4000-400 cm^{-1} region of (a) H-MOR and (b) H-SSZ-13 zeolites saturated sequentially with CH_3COCl doses at 298 K. The grey line is the spectrum of adsorbent-free H-MOR (Si/Al=10) and H-SSZ-13 (Si/Al=11.5) zeolites. The intensity of spectrums in H-SSZ-13 was doubled to better visualize the observed effects**.**

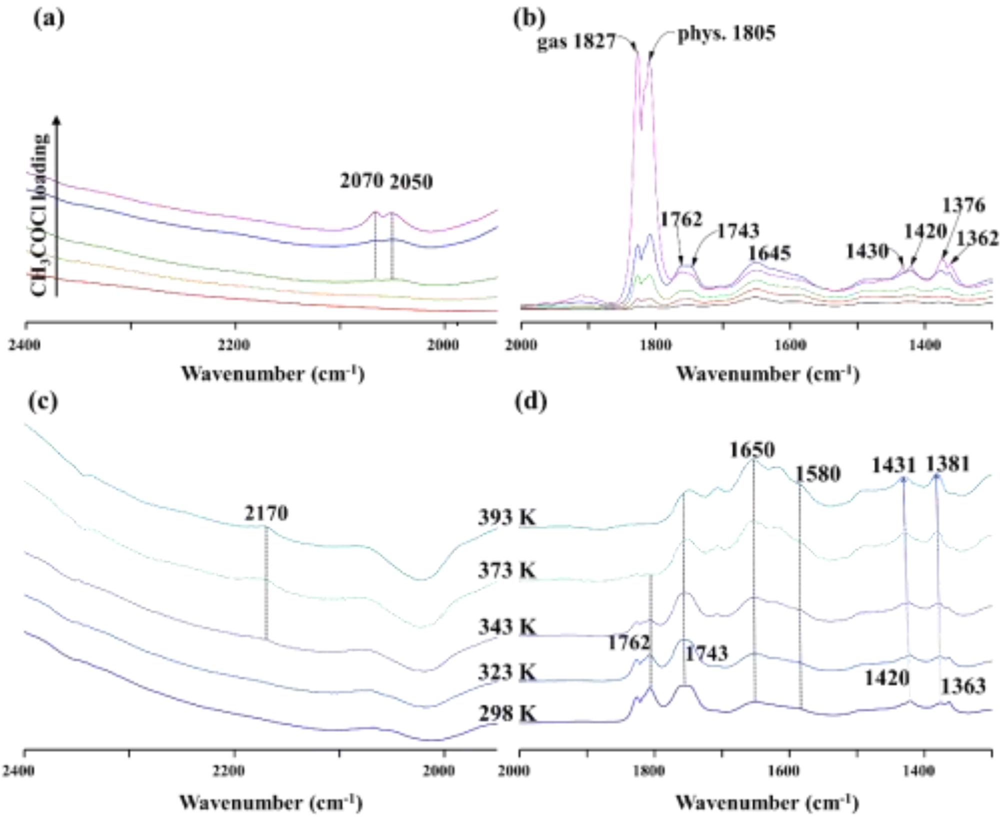

Figure S8. FT-IR spectra of CH_3COCl collected for (b and c) different loadings in H-SSZ-13 zeolite at (d and e) at different temperatures, both presented in (b and d) 2400 ~ 2000 cm^{-1}, (c and e) 2000 ~ 1300 cm^{-1} frequency regions. The intensity of spectra in H-SSZ-13 was doubled.

As shown the FT-IR spectra of the CH_3COCl adsorbed by H-SSZ-13 in Figure **S7b** and **S8a-S8b**, the bands in H-SSZ-13 were significantly weaker than these in H-MOR due to constrained diffusion of CH_3COCl in the cage-like pore structure of H-SSZ-13. Further, some bands in the C-O stretching vibrations region (2000~2400 cm^{-1}) have marginal intensity, if any. The band at 1645~1650 cm^{-1} was assigned to surface acetyl as consistent with that in H-MOR, (**Figure S8b**). The intensity of the new band at 2050~2070 cm^{-1} was enhanced with the increasing loading of CH_3COCl, which was attributed to the appearance of ketene species in H-SSZ-13 the most probably perturbed by the interaction with unreacted CH_3COCl and HCl. The ketene bands of similar frequencies like 2080 and 2052 cm^{-1} were observed also in H-MOR. When temperature increased to 323 K, the bands at 2080 and 2052 cm^{-1} were consumed in favor of the bands at 2170 cm^{-1}. Notably, the signal of acylium ion was not observed in H-SSZ-13 due to the lack of the confinement effect in CHA topology.

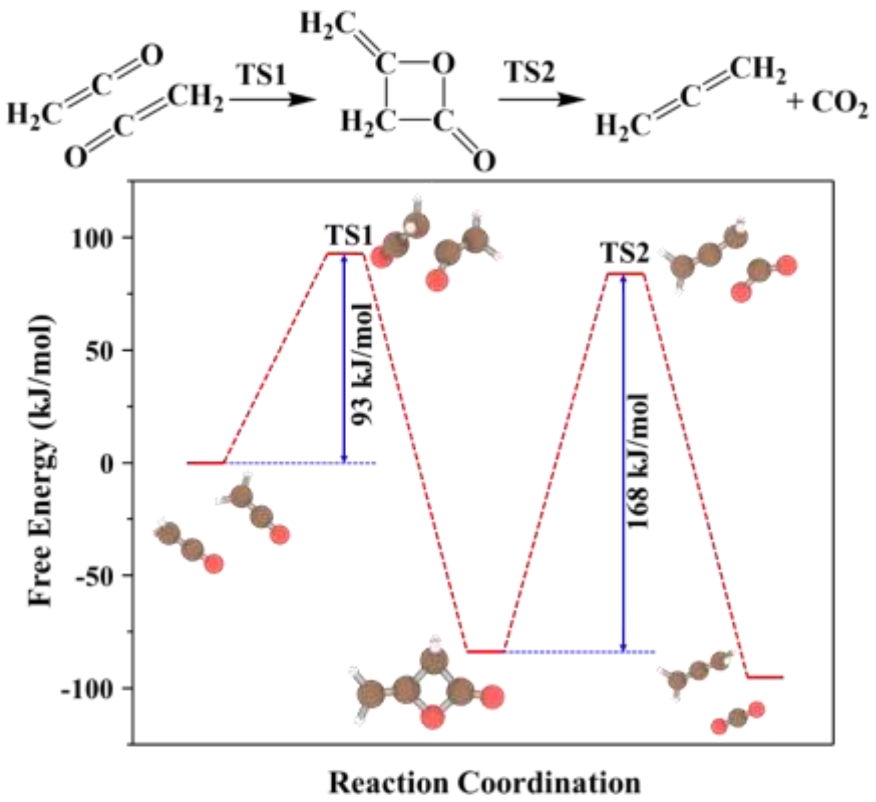

Figure S9. The reaction process of ketenes dimerization and further dissociation to CO_2 and propadiene in gas phase at 473 K by PBE-D3/6-31G(d, p) method.[S29]

In gas phase, ketene easily dimerize to diketene by overcoming a free energy barrier of 93 kJ/mol as a thermodynamic favorable process. The subsequent dissociation of diketene to propadiene and CO_2 is kinetically more limited than dimerization process since a free energy barrier of 168 kJ/mol has to be overcome. Notably, whole reaction path, *i.e.* from ketene to propadiene and CO_2, is thermodynamically feasible at 473 K, and these two free energy barriers would be further reduced under the confinement effect of zeolites. As the tautomer of propadiene, propyne is thermodynamically more stable species, it is rapidly formed by the isomerization of propadiene with the participation of Bronsted acid sites (BAS).

According to the isomerization of propadiene over a silica catalyst by Parmentier *et. al.*,[S30] the energy barrier of the isomerization from propadiene to propyne is only 54 kJ/mol under the catalytic effect of silanol. This energy barrier can be further decreased when the isomerization is catalyzed by either HCl or BAS of the higher acidic strength when the CH_3COCl is interacting with H-MOR. For this reason, the short-living diketene and propadiene intermediates cannot be observed in FT-IR spectra of CH_3COCl adsorbed in the zeolites even if the one FT-IR scan a performed within 0.20 sec. The most probably the higher scanning velocities have to be applied to detect these intermedies on the zeolite surface.

References:

[S1] C. Baerlocher, L. B. McCusker, *Database of Zeolite Structures*, 2013. http://www.iza-structure.org/databases.

[S2] T. Sun, W. Chen, S. Xu, A. Zheng, X. Wu, S. Zeng, N. Wang, X. Meng, Y. Wei, Z. Liu, The first carbon-carbon bond formation mechanism in methanol-to-hydrocarbons process over chabazite zeolite, *Chem*, **2021**, doi:10.1016/j.chempr.2021.05.023.

[S3] J. Hutter, M. Iannuzzi, F. Schiffmann, J. VandeVondele, cp2k: atomistic simulations of condensed matter systems, *Wires Comput Mol Sci*, **2014**, *4* (1), 15–25.

[S4] M. Bonomi, G. Bussi, C. Camilloni, G. A. Tribello, P. Banas, A. Barducci, M. Bernetti, P. G. Bolhuis, S. Bottaro, D. Branduardi, R. Capelli, P. Carloni, M. Ceriotti, A. Cesari, H. C. Chen, W. Chen, F. Colizzi, S. De, M. De La Pierre, D. Donadio, V. Drobot, B. Ensing, A. L. Ferguson, M. Filizola, J. S. Fraser, H. H. Fu, P. Gasparotto, F. L. Gervasio, F. Giberti, A. Gil-Ley, T. Giorgino, G. T. Heller, G. M. Hocky, M. Iannuzzi, M. Invernizzi, K. E. Jelfs, A. Jussupow, E. Kirilin, A. Laio, V. Limongelli, K. Lindorff-Larsen, T. Lohr, F. Marinelli, L. Martin-Samos, M. Masetti, R. Meyer, A. Michaelides, C. Molteni, T. Morishita, M. Nava, C. Paissoni, E. Papaleo, M. Parrinello, J. Pfaendtner, P. Piaggi, G. Piccini, A. Pietropaolo, F. Pietrucci, S. Pipolo, D. Provasi, D. Quigley, P. Raiteri, S. Raniolo, J. Rydzewski, M. Salvalaglio, G. C. Sosso, V. Spiwok, J. Sponer, D. W. H. Swenson, P. Tiwary, O. Valsson, M. Vendruscolo, G. A. Voth, A. White, Promoting transparency and reproducibility in enhanced molecular simulations, *Nat Methods*, **2019**, *16* (8), 670–673.

[S5] J. P. Perdew, K. Burke, M. Ernzerhof, Generalized gradient approximation made simple, *Phys. Rev. Lett.* **1997**, *77* (18), 1396–1396.

[S6] S. Grimme, J. Antony, S. Ehrlich, H. Krieg, A consistent and accurate ab initio parametrization of density functional dispersion correction (DFT-D) for the 94 elements H-Pu, *J. Chem. Phys.* **2010**, *132* (15), 154104.

[S7] G. Lippert, J. Hutter, M. Parrinello, The Gaussian and augmented-plane-wave density functional method for ab initio molecular dynamics simulations, *Theor. Chem. Acc.* **1999**, *103* (2), 124–140.

[S8] S. Goedecker, M. Teter, J. Hutter, Separable dual-space Gaussian pseudopotentials, *Phys. Rev. B* **1996**, *54* (3), 1703–1710.

[S9] T. J. Goncalves, P. N. Plessow, F. Studt, On the accuracy of density functional theory in zeolite catalysis, *ChemCatChem*, **2019**, *11* (17), 4368–4376.

[S10] P. N. Plessow, F. Studt, How accurately do approximate density functionals predict trends in acidic zeolite catalysis? *J. Phys. Chem. Lett.*, **2020**, *11* (11), 4305–4310.

[S11] Gesvandtnerova, M., Rocca, D., Bucko, T. Methanol carbonylation over acid mordenite: Insights from ab initio molecular dynamics and machine learning thermodynamic perturbation theory, *J. Catal.*, **2021**, *396*, 166–178.

[S12] Y. Chu, A. Y. Lo, C. Wang, F. Deng, Origin of high selectivity of dimethyl ether Carbonylation in the 8-membered Ring Channel of Mordenite zeolite, *J. Phys. Chem. C*, **2019**, *123* (25), 15503–15512.

[S13] H. A. Posch, W. G. Hoover, F. J. Vesely, Canonical dynamics of the Nosé oscillator: Stability, order, and chaos, *Phys. Rev. A* **1986**, *33* (6), 4253–4265.

[S14] A. Laio, M. Parrinello, Escaping free-energy minima, *Proc. Natl. Acad. Sci. U. S. A.* **2002**, *99* (20), 12562–12566.

[S15] I. Marcos-Alcalde, J. Setoain, J. I. Mendieta-Moreno, J. Mendieta, P. Gomez-Puertas, MEPSA: minimum energy pathway analysis for energy landscapes, *Bioinformatics* **2015**, *31* (23), 3853–3855.

[S16] M. Souaille, B. Roux, Extension to the weighted histogram analysis method: combining umbrella sampling with free energy calculations, *Comput. Phys. Commun.* **2001**, *135* (1), 40–57.

[S17] S. Kumar, J. M. Rosenberg, D. Bouzida, R. H. Swendsen, P. A. Kollman, The weighted histogram analysis method for free - energy calculations on biomolecules. I. The method, *J. Comput. Chem.* **1992**, *13* (8), 1011–1021.

[S18] G. Knizia, J. E. M. N. Klein, Electron flow in reaction mechanisms—revealed from first principles, *Angew. Chem. Int. Ed.* **2015**, *54* (18), 5518–5522.

[S19] G. Knizia, Intrinsic atomic orbitals: An unbiased bridge between quantum theory and chemical concepts, *J. Chem. Theory Comput.* **2013**, *9* (11), 4834–4843.

[S20] M. J. Frisch, G. W. Trucks, H. B. Schlegel, G. E. Scuseria, M. A. Robb, J. R. Cheeseman, G. Scalmani, V. Barone, B. Mennucci, G. A. Petersson *et al.*, Gaussian 09, Revision B.01, Gaussian, Inc., Wallingford CT, **2009**.

[S21] a) M. P. Mitoraj, A. Michalak, T. Ziegler, A combined charge and energy decomposition scheme for bond analysis, *J. Chem. Theory Comput.* **2009**, *5* (4), 962–975.

[S22] M. P. Mitoraj, A. Michalak, T. Ziegler, On the nature of the agostic bond between metal centers and β-hydrogen atoms in alkyl complexes. An analysis based on the extended transition state method and the natural orbitals for chemical valence scheme (ETS-NOCV). *Organometallics* **2009**, *28* (13), 3727–3733.

[S23] G. te Velde, F. M. Bickelhaupt, E. J. Baerends, C. F. Guerra, S. J. A. Van Gisbergen, J. G. Snijders, T. Ziegler, Chemistry with ADF, *J. Comput. Chem.* **2001**, *22* (9), 931–967.

[S24] E. Van Lenthe, E. J. Baerends, Optimized Slater - type basis sets for the elements 1 - 118, *J. Comput. Chem.* **2003**, *24* (9), 1142–1156.

[S25] D. B. Rasmussen, J. M. Christensen, B.Temel, F. Studt, P. G. Moses, J. Rossmeisl, A. D. Jensen, Reaction mechanism of dimethyl ether carbonylation to methyl acetate over mordenite–a combined DFT/experimental study, *Catal. Sci. Tech.*, **2017**, *7* (5), 1141-1152.

[S26] M. Boronat, C. Martínez, A. Corma, Mechanistic differences between methanol and dimethyl ether carbonylation in side pockets and large channels of mordenite, *Phys. Chem. Chem. Phys.*, **2011**, 13 (7), 2603-2612.

[S27] M. Boronat, C. Martínez-Sánchez, D. Law, A. Corma, Enzyme-like specificity in zeolites: a unique site position in mordenite for selective carbonylation of methanol and dimethyl ether with CO, *J. Am. Chem. Soc.*, **2008**, 130, 16316-16323.

[S28] N. Mehio, S. Dai, D. Jiang, Quantum mechanical basis for kinetic diameters of small gaseous molecules, *J. Phys. Chem. A* **2014**, *118* (6), 1150–1154.

[S29] R. J. Clemens, Diketene. *Chem. Rev.*, **1986**, *86* (2), 241-318.

[S30] J. H. Parmentier, H. G. Peer, L. Schutte. The isomerization of propyne into propadiene over a silica catalyst, *J. Catal.*, **1971**, *22* (2), 213-218.
